# Supplementary figures and images for: PbAP2-FG2 and PbAP2R-2 function together as a transcriptional repressor complex essential for Plasmodium female development
Source: PLoS Pathog. 2023 Feb 13;19(2):e1010890. doi: 10.1371/journal.ppat.1010890 (PMC9956629; doi:10.1371/journal.ppat.1010890)

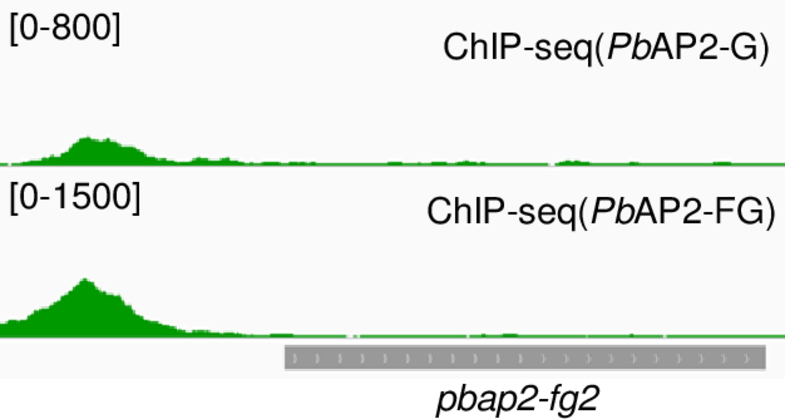

Supplement: S1 Fig — The grey bar indicates the gene body of pbap2-fg2. (TIF) [file ppat.1010890.s001.tif]

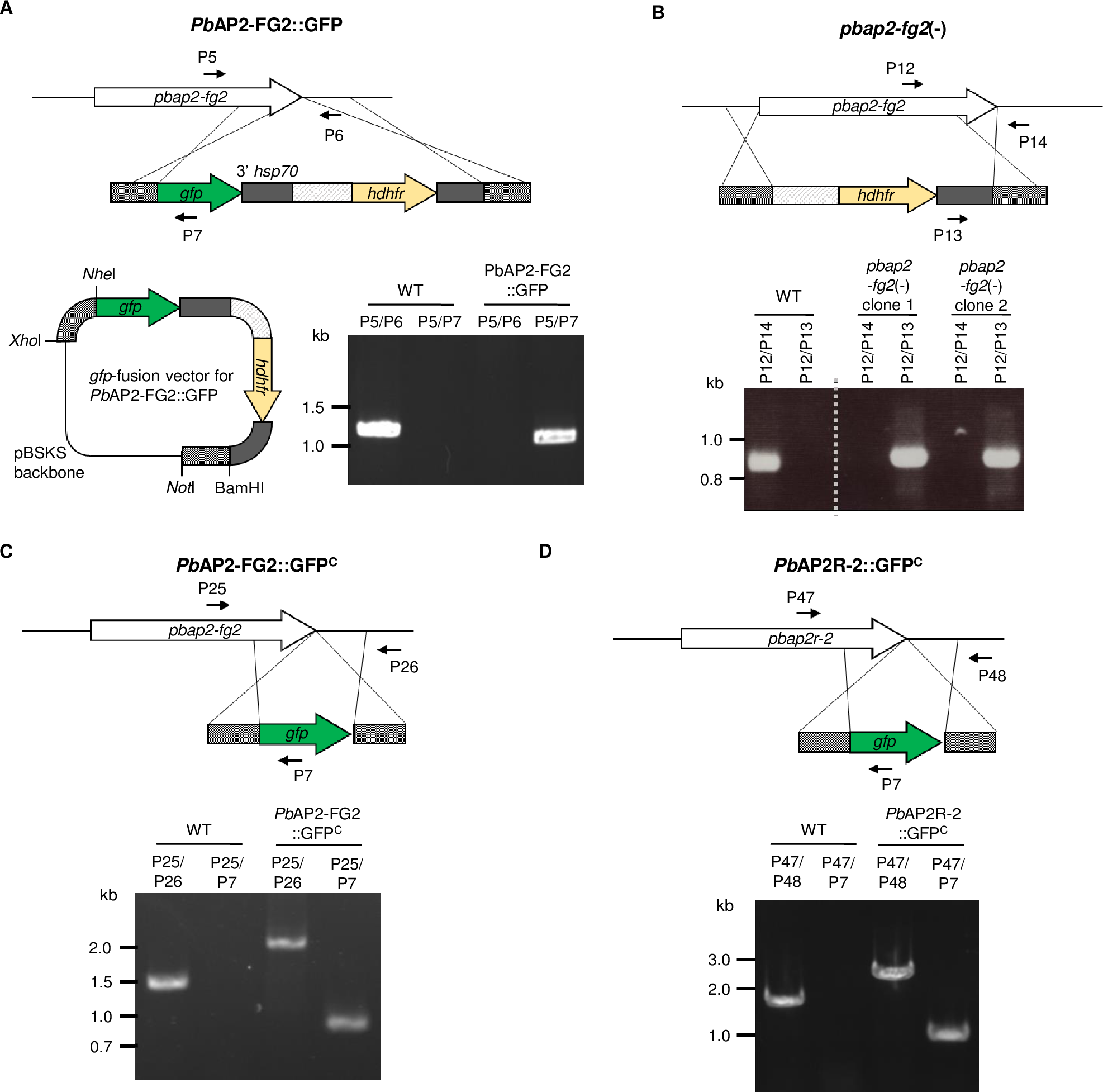

Supplement: S2 Fig — (A) PbAP2-FG2::GFP. (B) pbap2-fg2(-). (C) PbAP2-FG2::GFPC. (D) PbAP2R-2::GFPC. (TIF) [file ppat.1010890.s002.tif]

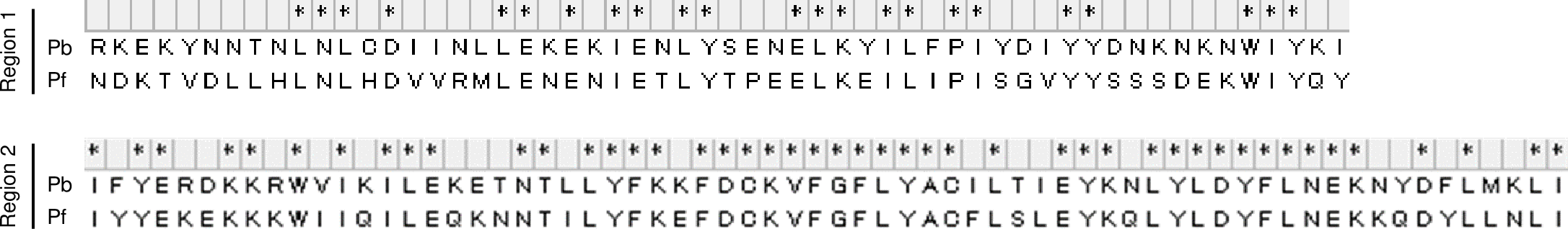

Supplement: S3 Fig — The sequences were aligned using the ClustalW program in Mega X. Asterisks indicate amino acids conserved between the two species. (TIF) [file ppat.1010890.s003.tif]

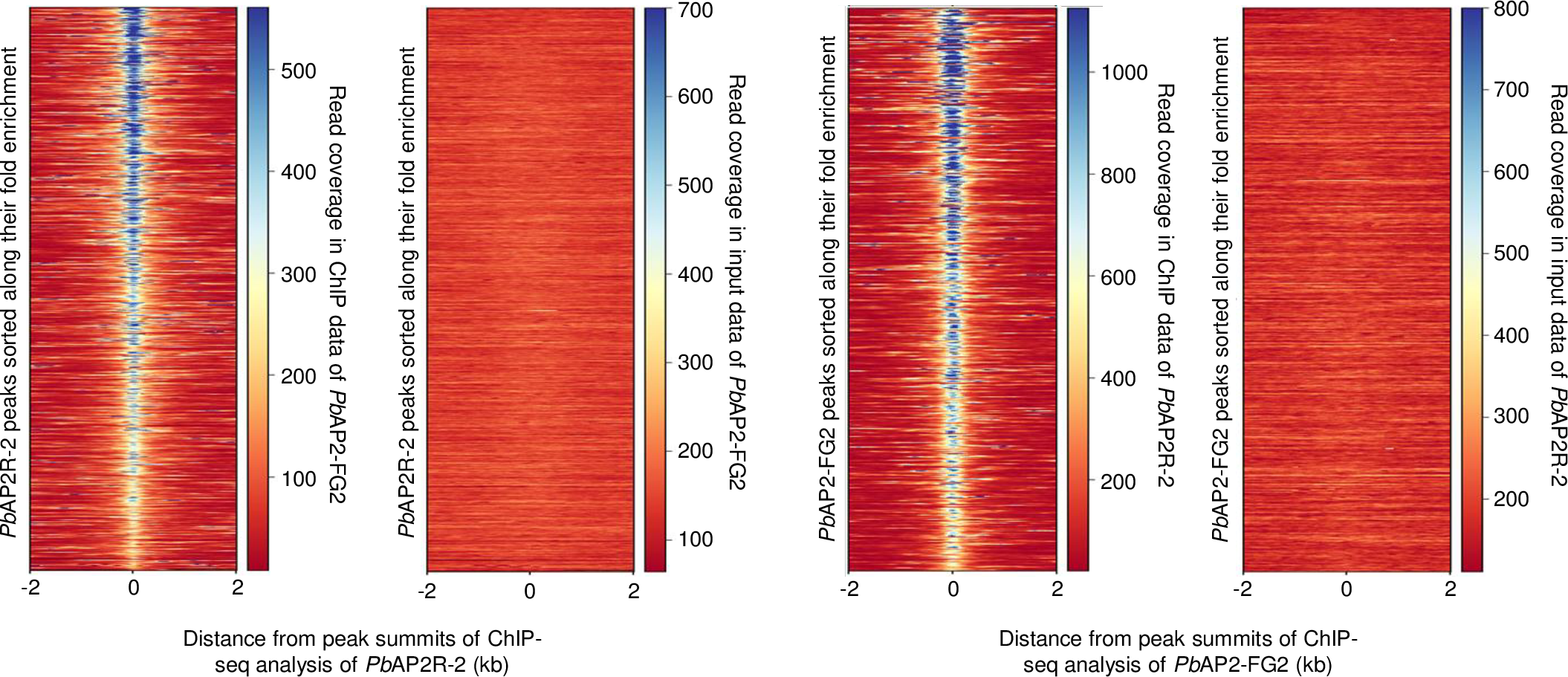

Supplement: S4 Fig — Peak regions are aligned in ascending order of their fold enrichment value. (TIF) [file ppat.1010890.s004.tif]
