# Supplementary material for: PbAP2-FG2 and PbAP2R-2 function together as a transcriptional repressor complex essential for Plasmodium female development
Source: PLoS Pathog. 2023 Feb 13;19(2):e1010890. doi: 10.1371/journal.ppat.1010890 (PMC9956629; doi:10.1371/journal.ppat.1010890)
Supplement: Attachment — Submitted filename: Responce_Letter.pdf [file ppat.1010890.s014.pdf]

12/27/2022

Responses to Reviewers:

We would like to thank you for taking your time to review our manuscript, titled "*Pb*AP2-FG2 and *Pb*AP2R-2 function together as a transcriptional repressor complex essential for *Plasmodium* female development." All of your comments were highly constructive and insightful, and thus, we sincerely made revisions according to these comments.

Part I - Summary

Reviewer #1: Male and female gametocytes are sexual progenitor cells essential for the transmission of the malaria parasite by the mosquito. Differentiation of gametocytes into fertile gametes depends on a sex-specific transcriptional program. How parasites establish different transcriptional repertoires in male and female gametocytes is still largely unknown. In this study, they discovered that the two female transcriptional regulators PbAP2-FG2 and AP2R-2 cooperate as a transcriptional repressor complex in P. berghei, with target genes including male, female, and early gametocyte genes activated by AP2-G. Based on their results, PbAP2-FG2 and AP2R-2 appear to play multiple roles in the development of female gametocytes from early gametocytes. This study was performed in the rodent model of P. berghei malaria and challenges a previous study of PyAP2-O3 (renamed PyAP2-FG2) in the P. yoelii model (reference 18, by Li et al.).

Thank you for your review, summary and all constructive comments. We sincerely revised our manuscript according to your comments.

Reviewer #2: In their manuscript: "PbAP2-FG2 and AP2-R2 function together as a transcriptional repressor complex essential for Plasmodium female development", Nishi et al. characterize the potential transcriptional regulatory role of two ApiAP2, DNA-binidngproteins, AP2R-2 and AP2-FG. Through phenotypic analysis it would seem the AP2-FG2 protein peaks in expression in female gametocytes and the genetic perturbation of this protein results in a decrease in female gametocyte-related genes. The protein seems to exert its function through binding upstream regions of genes, repressing male and female-related gametocyte genes alike. A similar profile was observed for AP2R-2. After thorough reexamination of previously published data for

the orthologue of AP2-FG2 in Plasmodium yoelli, the authors demonstrate significant overlap between the function of the two orthologous proteins. Overall, this study confirms that the role of the newly coined "AP2-FG2" in Plasmodium berghei is consistent to what has previously been published for Plasmodium yoelii. I thank the authors for a well organized, and clearly communicated study. However, there are both major and minor concerns with this manuscript and it is requested that the authors respond to each and make edits where appropriate to strengthen the manuscript.

Thank you for your review, summary and all constructive comments. Especially, those comments regarding our statistical analyses and methodologies were very helpful. We sincerely revised our manuscript according to your comments.

Reviewer #3: The gametocyte stage is a critical step to continuing the Plasmodium lifecycle from the vertebrate host to mosquito vector. However, the fine-tuned regulation of this life stage transition is not completely understood. This manuscript focuses on PbAP2-FG2 and PbAP2R-2, which are two putative female gametocyte regulators. PbAP2-FG2 (for "female gametocyte 2") was previously named PbAP2-O3 (PBANKA_1015500; "ookinete 3") in Modryznska et al. (2017) Cell Host & Microbe due to the ap2-o3 KO causing a growth arrest prior to the ookinete stage of mosquito development. In this submission, the authors have renamed the factor to PbAP2-FG2, due to expression in female gametocytes and dysregulation of female gametocyte maturation genes after PbAP2-FG2 disruption. The second factor, PbAP2R-2 (stands for "AP2-related factor 2") was named in previous work by this group in Yuda et al. (2021) Parasitology International because it contained an ACDC domain and was a target of PbAP2-G and PbAP2-FG via ChIP-seq. In this work, Nishi et al. employed a variety of approaches to identify the role of both PbAP2-FG2 and PbAP2R-2, including (1) GFP-tagging PbAP2-FG2 that identified peak protein expression during female gametocyte development, (2) gene KO of PbAP2-FG2 coupled with RNA-seq that identified an enrichment of female-specific dysregulated transcripts over male-specific transcripts, (3) anti-GFP chromatin immunoprecipitation followed by sequencing (ChIP-seq) of PbAP2-FG2 that identified its genome-wide binding sites and enriched RGAGAR DNA motif, (4) integration of ChIP-seq gene targets and RNA-seq DEGs that identified an enrichment of ChIP-seq targets with the female-specific genes upregulated, (5) CRISPR-Cas9 modifications to remove the RGAGAR motif from a promoter region that identified a depletion of PbAP2-FG2 binding by ChIP-qPCR, (6) CRISPR-Cas9 to integrate a GFP tag onto the 3`-end of pbap2r-2 and an anti-GFP

ChIP-seq that identified the binding sites of PbAP2R-2, which had a high overlap with PbAP2-FG2 ChIP-seq binding sites, (7) integration of PbAP2-G ChIP-seq targets that identified many gene targets of PbAP2-G as targets of PbAP2-FG2 and PbAP2R-2, and finally (8) a re-analysis of ChIP-seq data from Li et al. (2021) EMBO Reports on the PbAP2-FG2 P. yoelii ortholog, PyAP2-O3, where they identified their enriched RGAGAR motif in the highly reproducible ChIP-seq binding sites.

Overall, the results presented in this manuscript regarding PbAP2-FG2 are exciting and the data analysis is thorough. The results provide a new characterization of an ApiAP2 protein PbAP2-FG2 involved in the regulation of gametocyte development, particularly in female cell fate determination. However, despite a number of preliminary results, the suggestion that the data presented demonstrate a transcriptional regulatory complex between PbAP2-FG2 and PbAP2R-2 that work together to repress gene targets is unsubstantiated. The data clearly show that PbAP2-FG2 and PbAP2R-2 target highly similar genomic regions, but proof that those components are physically interacting in the same cell at the same time are lacking.

Thank you for your review, summary and all constructive comments. Also, thank you for addressing grammatical errors and word choices in the manuscript. That was really helpful. We sincerely revised our manuscript according to your comments.

Part II – Major Issues: Key Experiments Required for Acceptance

Reviewer #1: 1- I found quite puzzling that there few if no correlation between the direct targets of PbAP2-FG2 (assessed by ChIP-seq) and the dysregulation of the transcriptome in its absence. The authors have attempted to explain these discrepancies, but their hypothesis is elusive and does not allow for the possibility that this factor could be replaced, for example, by another ApiAP2. Is it possible that each AP2 they are describing homodimerizes individually and compensates for the absence of the other.

Thank you for the comment. Upregulation of a gene could be harder to be detected than downregulation because a level of upregulation in the absence of a repressor depends on a transcriptional activator that activates the gene. In addition, if a gene was expressed at a substantial level in WT, its upregulation in a knockout parasite becomes harder. This

could happen especially when RNA-seq analysis was performed with mixed populations (in our case, early, male, and female gametocytes). Nevertheless, we were still able to demonstrate that the overall correlation between the target genes of *Pb*AP2-FG2 and the genes upregulated in *pbap2-fg2*(-) was virtually significant as shown by Fisher's exact test (Line 264-266) and Student's t-test (Line 269-272).

In *Plasmodium*, it is unlikely that two transcription factors play the same role on the same *cis*-regulatory element at the same cell stage independently, because *Plasmodium* parasites have only a small number of sequence-specific transcription factors on the genome. In addition, even if a transcription factor that functions just as *Pb*AP2-FG2 exists, this factor does not have ability to fully compliment the loss of *pbap2-fg2* (disruption of *pbap2-fg2* or *pbap2r-2* alone impairs female development). Existence of such a factor seems very redundant, especially for organisms with a limited number of sequence-specific transcription factors on their genome. Therefore, we do not include such possibility in our discussion unless something that strongly suggests such possibility comes up. For *Pb*AP2-FG2 and *Pb*AP2R-2, their interaction was proven by the RIME, thanks to the suggestions made by all three reviewers. Therefore, the possibility that these two factors compensate each other can be ruled out.

2- With their elegant experiments on cis-mutagenesis of the repressive element, they provide only a partial answer to the lack of correlation at the genomic level between ChIPseq and RNAseq by proposing that PbAP2-FG2 and PbAP2-R2 cooperatively repress genes, probably as heterodimers. However, the possibility that both homodimerize and colocalize at the same genomic sites cannot be ruled out until it is shown that they interact biochemically or that PbAP2-R2 is released from chromatin in the absence of PbAP2-FG2 and vice versa.

Thank you for your comment. As you mention, our results were insufficient to conclude that *Pb*AP2-FG2 and *Pb*AP2R-2 form a heterodimer complex. Considering your comment, we performed ChIP followed by MS (rapid immunoprecipitation mass spectrometry of endogenous proteins; RIME) using the *Pb*AP2-FG2::GFP parasite to evaluate whether the two factors actually form a complex. Through the analysis, we revealed that *Pb*AP2R-2 was coimmunoprecipitated with *Pb*AP2-FG2, proving the formation of a complex between them. Besides *Pb*AP2R-2, we also detected other proteins that might be involved in transcriptional repression by the RIME. We described these results in the Results (Line 404-436) and Discussion (Line 612-641) sections. Also, we described the RIME method in the Materials and Methods section (Line 788-826).

Reviewer #2: 1. Secondarily, the authors abandon any notion that this protein has a role in any other stage of development quite quickly, despite it's obvious expression in both male gametocytes and ookinetes. Could the authors clarify why neither of these were pursued further? Is it clear to them that there is no possibility of a role for this AP2 in other stages of development, why or why not? In addition, please quantify all of the phenotypes that are suggested by both the GFP-tagged and genetic disruptants of AP2-FG2

Thank you for the comment. As we described in the Results, we only detected expression of *Pb*AP2-FG2 in female gametocytes of *Pb*AP2-FG2::GFP. The parasites at the other stages exhibited no obvious fluorescence especially in their nucleus as shown in Fig 1B. This result was consistent with the previous studies in *P. yoelii*. Therefore, we did not think that further analysis was needed for the role of *Pb*AP2-FG2 in male gametocytes or after fertilization. Meanwhile, for male gametocyte stage, we showed that exflagellation in *pbap2-fg2*(-) was normal (we provided an appropriate data as Fig 1D in the revised manuscript following your suggestion below), and the cross-fertilization assay revealed that the male gametocyte of *pbap2-fg2*(-) was fertile. For the possible role of *Pb*AP2-FG2 during mosquito stages, we were not able to perform the phenotype analysis because *pbap2-fg2*(-) is not able to produce any oocyst. We might assess the role of *Pb*AP2-FG2 during the mosquito stages using conditional knockdown/knockout methods in the future studies.

2. The authors have completed a tremendous amount of work to characterize these proteins and it will add to the already large number of ApiAP2s this lab has revealed the role of. They are to be commended for their continued efforts. However, to support the assertion that AP2-FG2 and AP2R-2 act together to co-repress genes, the authors must provide definitive data that demonstrate an interaction between the two lines. The authors should consider Co-IP or tagging AP2-FG2 with 3xHA in a parasite that has AP2R-2 tagged with GFP. Flow-cytometry can be used to select for parasites expressing AP2R-2 and ChiP-seq for either or both AP2 can be performed.

Thank you for your suggestion. As you comment, our experiments were not enough to prove formation of a complex between *Pb*AP2-FG2 and *Pb*AP2R-2. Therefore, we performed ChIP followed by MS (rapid immunoprecipitation mass spectrometry of endogenous proteins; RIME) using the *Pb*AP2-FG2::GFP parasite to confirm whether the

two factors actually form a complex. The result showed that *Pb*AP2R-2 was coimmunoprecipitated with *Pb*AP2-FG2, proving the formation of a complex between them. Besides *Pb*AP2R-2, we also detected other proteins that might be involved in transcriptional repression by the RIME. We described these results in the Results (Line 404-436) and Discussion (Line 612-641) sections. Also, we described the RIME method in the Materials and Methods section (Line 788-826).

Reviewer #3: There is insufficient data presented to demonstrate that there is a complex formed between PbAP2-FG2 and PbAP2R-2 implying colocalization. Therefore, the title and many statements in the Results and the beginning and end of the Discussion need to be modified to reflect this. Although the authors do demonstrate that the PbAP2-FG2 and PbAP2R-2 appear to occupy similar genomewide binding sites, they do not test whether this is due to complex formation. At least two possibilities come to mind. It is entirely feasible that in 2 different cell populations PbAP2-FG2 is binding these loci throughout the genome, while in a different population PbAP2R-2 is binding the same loci. This can only be resolved by single cell ChIP-seq which has not been successful to date in Plasmodium parasites (to my knowledge). Although this is unlikely given the roughly 1:1 correlation of the ChIP-seq peak intensities reported, it is possible. To directly test whether there is a complex, immunoprecipitation (IP) experiments will need to be performed. Both PbAP2-FG2 and PbAP2R-2 were tagged in this manuscript and used for localization studies and ChIP-seq. Therefore, IP-MS proteomics should be feasible in principle. To claim that PbAP2-FG2 and PbAP2R-2 form a complex, the authors should produce IP/MS data using all available tagged reagents already at their disposal.

Thank you for your suggestion. As you comment, our statement about formation of a complex between *Pb*AP2-FG2 and *Pb*AP2R-2 was based on insufficient data. Taking your advice, we performed ChIP followed by MS (rapid immunoprecipitation mass spectrometry of endogenous proteins; RIME) using the *Pb*AP2-FG2::GFP parasite to confirm whether the two factors actually form a complex. The result showed that *Pb*AP2R-2 was coimmunoprecipitated *Pb*AP2-FG2, proving the formation of a complex between them. Besides *Pb*AP2R-2, we also detected other proteins that might be involved in transcriptional repression by the RIME. We described these results in the Results (Line 404-436) and Discussion (Line 612-641) sections. Also, we described the RIME method in the Materials and Methods section (Line 788-826).

Throughout the manuscript (e.g. Line 73, Line 533), reference to P. berghei targets of AP2-G are mentioned solely in the context of previous work by the same authors, However, other studies have determined targets of PfAP2-G by inducible activation, ChIP-seq, or scRNA-seq including Llora-Battle et al. 2019, Josling et al. 2020, and Poran 2017. Two points are worth addressing. First, what is the overlap in predicted targets between these studies? Can the authors speculate why they are different between PbAP2-G (many targets) and PfAP2-G (limited target set)? For example, the authors previously reported pbap2-g2, pbap2-fg, and pbab2-z as targets of PbAP2-G. However, according to the literature this is not the case for PfAP2-G, where these other ApiAP2 genes are not direct targets.

Thank you for the comment. As you suggest, target genes of *Pf*AP2-G and *Pb*AP2-G identified by the ChIP-seq analyses are largely different. In the manuscript, we only focused on the targets of *Pb*AP2-G since the study was conducted using *P. berghei*. To clarify that, we used the full ID (*Pb*AP2-G) throughout the manuscript (following your advice in Minor issues). We agree with your suggestion that the reason for the difference between the target genes of *Pf*AP2-G and *Pb*AP2-G is worth addressing. We speculate that this difference largely derives from a difference in duration of gametocyte development between the two species. However, we also think that it does not seem appropriate to discuss about the difference in the target genes of *Pf*AP2-G and *Pb*AP2-G because these data were previously reported and not a main data in this manuscript.

The study that performed inducible activation of *Pb*AP2-G also proposed putative targets of *Pb*AP2-G. Of these, the genes induced within 6 h after induction of *Pb*AP2-G were significantly overlapped with the target genes of *Pb*AP2-G identified by the ChIP-seq as described in our previous study. This result confirmed accuracy of our target analysis for *Pb*AP2-G. Meanwhile, as you commented below in the section of Minor issues, RNA-seq alone is not able to identify the direct targets of a transcriptional regulators. Therefore, as the target genes of *Pb*AP2-G, we only used our previous data, which was based on the ChIP-seq of *Pb*AP2-G.

Next, overall the manuscript is highly under-cited. Please be more inclusive in recognizing the contributions of others. One major omission in the Introduction is reference to the recent work of Russell et al. who have clearly demonstrated roles for a helix-turn-helix protein and other zinc-finger proteins in the regulation of male and female sexual development lineages of Pb parasites: BioRxiv: 2021.08.04.455056v1 This study is highly relevant to the current work and an understanding of sexual

maturation and sex lineage differentiation.

We are sorry for the poor citation in the manuscript. We thoroughly revised our manuscript and previous studies and added several studies in the Reference.

About the work you mention, we were actually aware of it and thought that the study showed remarkable results by creative methods. However, we were not sure if it was appropriate to cite a study that is still in a preprint state in the Introduction. Since you recommended us to cite the study, we decided to describe the study in the Results section as follows;
Line 348-349. "Furthermore, in the recent study by Russell et al., *ap2r-2* was identified as one of the genes critical for female differentiation (still in a preprint [45])."

Given these potential species-specific differences, it would be worthwhile to identify each ApiAP2 discussed with the relevant species designation (Pb, Py, Pf) based on where the evidence presented originates. There are clear reasons to believe that target genes in one organism may or may not be the same across species. Similarly, it has already been demonstrated that certain homologous ApiAP2 proteins are essential in some species, but not all. By definition, their functional roles must therefore be different or there is functional redundancy in one species. Therefore, please use full IDs throughout such as PbAP2-G2 or PbAP2R-2 or PfAP2-G2, etc…

Thank you for your suggestion. We found your opinion about the potential species-specificity of AP2 proteins in *Plasmodium* very important. We assigned species designation to all AP2 genes/proteins described in the manuscript according to your comment.

Another clear issue is comparing the results in lines 389-294 between Pf and Pb and the role of the cytoskeleton. Clearly the timing and duration of these complexes must be vastly different given the 24-hour maturation of gametocytes in Pb versus 10 days in Pf.

Thank you for your comment. As you suggest, cytoskeletal development during gametocyte development of *P. berghei* and *P. falciparum* should be considerably different. We are sorry that our statement did not clearly separate these two species in this context. We did not mean to compare the two species in this section. What we really wanted to suggest was just that cytoskeleton-related genes could play a role in early gametocyte

development but not during female maturation in *P. berghei*. Meanwhile, to suggest that the complex is widely used in the *Plasmodium* life cycle, we decided to cite the studies of *Pf*TRiC because these were the only studies of TRiC in *Plasmodium* species. To clarify, we rewrote the statement as follows;
Line 463-469. "Thus, this complex is presumed to be widely required in the *Plasmodium* life cycle, which, for *P. berghei*, includes early gametocyte development, but not during female development. Notably, the common target genes of *Pb*AP2-FG2 and *Pb*AP2-G also included the actin I gene, alpha-tubulin 2 gene, and a putative tubulin beta chain gene (S3C Table). These findings suggested the possibility that for female gametocytes of *P. berghei*, cytoskeletal development is mostly completed during early gametocyte development."

In generating tagged parasite lines for this study (Figure 1C) it is unclear if the endogenous 3`UTR is maintained in these parasites. This same group reported that altering the endogenous 3' UTR was found to be an issue in previous work on AP2-Z (Yuda et al. (2021) and Nishi et al. (2022) BioRxiv). This was reportedly because the replacement of the endogenous 3`UTR disrupted the normal function of DOZI and the translational repression of AP2-Z. Please label the plasmid designs in Fig S1 more clearly and please note any changes to the 3`UTR in the main text.

Thank you for the notice. We are sorry that the design of *Pb*AP2-FG2::GFP was not clear in the manuscript. The 3' UTR of *gfp*-tagged *pbap2-fg2* in *Pb*AP2-FG2::GFP was not the endogenous 3' UTR of *pbap2-fg2* but that of *hsp70*. To clarify this parasite design, we modified S2A Fig (S1A Fig in the previous manuscript) and rewrote the Materials and Methods as follows;
Line 681-685. "The *gfp*-fusion vector was previously generated on the backbone of pBluescript SK (+) (Stratagene, La Jolla, CA, USA). In this vector, 3' untranslated region (UTR) of *hsp70* and a *hdhfr* expression cassette (under the control of *pbef-1α* promoter and 3' UTR of *pbdhfr*) were located 3'-side of the *gfp* gene in this order."

Thank you for notifying our previous works and importance of keeping endogenous 3' UTR for female-transcribed genes. However, we did not focus on the replacement of 3' UTR in the case of *Pb*AP2-FG2::GFP because in the later section of the manuscript, we also developed *Pb*AP2-FG2::GFP^{C}, in which the endogenous 3' UTR of *pbap2-fg2* was retained, using the CRISPR/Cas9 system. This parasite line also showed nuclear-localized GFP signal in the female gametocytes, and the ChIP experiments were successful using the gametocytes of *Pb*AP2-FG2::GFP^{C}. Therefore, we considered that it

was clear that *pbap2-fg2* is not translationally repressed in females.

The authors use a nice cross-fertilization assay to demonstrate the role of pbap2-fg2. The same should be done for pbap2r-2, and the tools to do so are available.

Thank you for your suggestion. We performed a cross-fertilization assay between *pbap2r-2*(-), *p47*(-), and *p48/45*(-) as well and described the result as follows;
Line 351-357. "First, we performed a cross-fertilization assay to confirm the role of *Pb*AP2R-2 in female gametocyte development. When crossed with *p47*(-), *pbap2r-2*(-) could not produce normal ookinetes, confirming that development of female gametocytes in *pbap2r-2*(-) was impaired (Fig 6A). On the other hand, when crossed with *p48/45*(-), *pbap2r-2*(-) produced mature ookinetes nearly as many as when *p47*(-) and *p48/45*(-) were crossed (Fig 6A). These results revealed that as suggested by the previous studies, *Pb*AP2R-2 is essential for female development."

To determine differentially expressed genes (DEGs), the authors reference "previously reported sex-specific RNA-seq data" from reference 21 (lines 168, 169). This choice appears arbitrary. There are numerous datasets available (transcriptomic, proteomic, genomic) that have catalogued putative male and female gametocyte markers. A much better approach would be to use a comprehensive table with multiple lines of evidence to categorize individual genes as "male" or "female", especially since most of them have not been independently characterized. Further, these genes should be referred to as "male-enriched" and "female-enriched" as opposed to "male-specific" or "female-specific", because few genes are only truly present in one lineage.

Thank you for your suggestion. For defining sex-enriched genes, we only focused on transcriptome data because we were assessing the roles of *Pb*AP2-FG2/*Pb*AP2R-2 in transcriptional regulation during gametocyte development. In this case, proteome data is not appropriate to define sex-specific genes because majority of female enriched genes are translationally repressed in female gametocytes. In addition to the data presented by Witmer et al., there is another sex-specific transcriptomic data, which has been presented by Lee et al. 2017. We first thought that combining the data from the two studies could be a better way to define sex-enriched genes as you suggest. However, as we assessed the list of genes that are enriched in male compared to asexual in the study by Lee et al. (additional file 3), we found that the list contained many female genes, including *ccp2* and *p28*. Hence, we suspected that quality of the data might not be very good. In addition,

Witmer et al. used the non-gametocyte producer parasite line to prepare the "asexual" transcriptome data. Therefore, we considered that the data presented by Witmer et al. 2020 was better and decided not to combine the two data.

Since you suggested, we realized that in most cases, "female-enriched" and "male-enriched" are more appropriate to describe a gene/protein expression property than "female-specific" and "male-specific". Therefore, we rephrased them accordingly. Meanwhile, we kept the phrase for referring to *pbap2-fg2*, *pbap2r-2*, *ap2-fg*, and dynein heavy chain gene (PBANKA_0416100) as they are in fact "female-specific" and "male-specific", respectively.

The ChIP-seq results are quite surprising with over 1000 peaks in every replicate experiment. Compared to other ChIP-seq results in the Plasmodium literature, this number is very high for an individual DNA binding protein. Looking back, virtual all papers from this group report high numbers of binding events. What number of genes does this represent? Presumably not 1/5 of the genome? If not, does the appearance of multiple binding events/motifs upstream of genes suggest something associated with regulation?

Thank you for the comment. About the number of peaks detected in ChIP-seq, we think over 1000 is not very surprising in the studies of *Plasmodium*. Besides our studies of AP2 transcription factors, other studies of *Plasmodium* transcriptional regulators, such as *Py*AP2-O3 and *Pf*HDP1, have reported over 1000 peaks, and the study of *Pf*AP2-G2 even detected nearly 5000 peaks in their ChIP-seq analysis. As you mention, the target genes predicted from those ChIP-seq include a large part of the genome, which indicates that transcriptome of each cell stage can be regulated by a few *cis*-regulatory elements. As we have discussed previously, such a simple regulation enables the parasite to complete the life cycle with a small number of sequence-specific transcriptional regulators on the genome (in our opinion).

Due to the high overlap in binding sites between PbAP2-FG2 and PbAP2R-2, the authors should check whether those sites are also pulled down when performing an anti-GFP ChIP-seq in a WT parasite background (i.e., are those sites hyper ChIP-able)?

Thank you for your comment. We understand that readers might concern about technical causes, such as hyper ChIPable regions when they see a significant overlap between

ChIP-seq analyses for two different proteins. However, we have been performing ChIP-seq experiments for several *Plasmodium* proteins, including *Pb*AP2-G and *Pb*AP2-FG, during the gametocyte stage using the same methods and the same anti-GFP antibody and have obtained quite distinct results for each of these proteins. In addition, none of these ChIP-seq analyses detected the GAGA-related motifs as an enriched motif. Therefore, we are sure that those peaks highly overlapped between the ChIP-seq of *Pb*AP2-FG2 and *Pb*AP2R-2 are not derived from non-specific background or hyper ChIPable regions and do not think that further investigation is needed to prove it.

Also, how many of the more than one thousand binding sites have the motif reported in Figure 3C. It is not clear. Please prepare a figure showing the peak summits for all binding events colored by the underlying nucleotide sequence.

As mentioned in Line 223-226, “85% of the peaks had at least one of these enriched motifs within 300 bp of the summit. Moreover, for more than half of these peaks, the distance between the peak summit and the nearest motif was within 50 bp (Fig 3D)”. To make the result clear in the figure, we added percentage of peaks that have the motifs in Fig 3C. We think that with this modification, the Fig 3C and 3D sufficiently demonstrate the relationship of peak regions and the enriched motifs; Fig 3C shows significance of enrichment for each motif and ratio of peaks containing the motifs, and Fig 3D shows distance between peak summits and the nearest motifs. Therefore, about the figure you suggest, we are not sure if it must be presented because it does not seem to show any new aspect of the data that has not been shown by Fig 3C and 3D.

The statement in line 320-321 that AP2-R2 has no AP2 domain is unsubstantiated. What is this evidence based on? The few descriptions in the literature of the ACDC domain suggest that it must always co-occur with an AP2 domain. In fact, in the Pf orthologue (PF3D7_1319600), Oehring et al. 2012 (Figure 8) identified 2 AP2 domains in this “ACDC domain-containing protein”. Please provide an alignment in the Supplementary materials between the Pb and Pf orthologues for AP2R-2. Also, using AlphaFold2 to examine the predicted structure of AP2R-2 clearly demonstrates at least one well-folded AP2 domain and perhaps a cryptic second AP2 domain. The authors should provide this information. In addition to demonstrating that this protein may have 1-2 AP2 domains, it may also provide an alternative interpretation of why the binding of AP2R-2 and AP2-FG2 are so similar? Perhaps their AP2 domains are actually relatively similar and therefore bind the same motif and genome-wide sites?

<u>Otherwise, it is also unclear why the authors pursue ChIP-seq of AP2R-2 if they do not suspect that it has any DNA binding domains.</u>

Thank you for your comment. As you mention, the study by Oehring et al 2012 indicated that *Pf*AP2R-2 has two AP2 domains which were found through HMMER search. However, they did not indicate the amino acid sequences (or positions) that were detected as "AP2" in their manuscript, which made it very difficult for us to assess if the domains are conserved and annotated as "AP2" in the other *Plasmodium* species as well. As we performed the domain search, we did not find any AP2 domain in AP2-FG2 by SMART, Pfam, or even HMMER (hmmscan) for both *P. berghei* and *P. falciparum*. In addition, through secondary structure prediction by PSIPRED, no region with obvious three-stranded antiparallel beta-sheets and one alpha-helix, which are the common secondary structural feature of AP2, was detected within the amino acid sequence of AP2R-2. Alignment of amino acid sequences of *Pb*AP2R-2 and *Pf*AP2R-2 showed two highly conserved regions, one of which was the ACDC domain. The other region showed 84% conservation of amino acids (32/38 amino acids, position 1220-1257 for *Pb*AP2R-2) between the two species and are consisted of a beta-sheet and an alpha-helix. With the N-terminal adjacent region, we predicted 3D structure of this conserved region (position 1202-1257), which resulted in a structure similar to AP2 with the first beta-sheet not formed. However, the 3D structure was not found similar to any AP2 by VAST search, and this region did not fully contain consensus amino acids for AP2. In addition, the first and second beta-sheet, which is essential for DNA-binding, were not highly conserved between *Pb*AP2R-2 and *Pf*AP2R-2. Therefore, we thought that this conserved region might have been an AP2 domain in ancestral species but may not be functional as a sequence-specific DNA binding domain in the present *Plasmodium* species.

Meanwhile, we now agree with your suggestion that it is not appropriate to just state "*Pb*AP2R-2 has an ACDC domain at its C-terminus, but no AP2 domain." We therefore changed the statement as follows;

Line 340-346. "*Pb*AP2R-2 has an ACDC domain at its C-terminus. A previous study indicated that *Pf*AP2R-2 has two putative AP2 domain [42], one of which is not conserved in the *Plasmodium* species. The other one had AP2-like structure in both *P. berghei* and *P. falciparum*, but only the third beta-sheet and the alpha-helix were highly conserved between the two species. Because three-stranded antiparallel beta-sheets in AP2 are the structure that interacts with DNA [43,44], it was considered that this AP2-like domain is probably not involved in binding to DNA of a specific sequence."

<u>In the re-analysis of the PyAP2-FG2 ChIP-seq data from Li et al. please describe clearly how your methods of analyzing the ChIP-seq data differ. You describe how you have re-analyzed their data, but have you re-analyzed it using their approach? And which approach is better and why beside the inclusion/exclusion of low read counts?</u>

Thank you for your comment. For the reanalysis of the ChIP-seq data of *Py*AP2-O3, the methods used in our analysis and in the *Py* study were very similar; we used bowtie2 to map the sequence data while Li et al. used bowtie. Peaks were called with macs2 in both approaches. For the threshold of peaks, we tested both their criteria and ours as described in the Results section. Although we think that results produced by bowtie2 and bowtie are not largely different, it is said that when dealing with reads longer than 50 bp, bowtie2 is generally superior to bowtie. For the ChIP-seq analysis, important difference was the motif enrichment analysis.

About "inclusion/exclusion of low read counts", we did not make such change in the ChIP-seq analysis but set a threshold for FPKM in the RNA-seq analysis. Thus, we assumed that you meant to ask about reanalysis of the RNA-seq data as well. For the RNA-seq data, we used hisat2, featureCounts, and DESeq2 as described in the Materials and Methods. On the other hand, Li et al analyzed the data as follows. First, bowtie2 was used for mapping reads to rRNA database to remove the rRNA-mapped reads. The remaining reads were then mapped to reference genome by tophat2. Gene abundances were quantified by software RSEM. For the differential expression analysis, the edgeR package was used. Trimmed mean of M-values normalization (TMM) method was used, and the gene expression level was normalized by using FPKM method. Genes with a fold change ≥ 2 and a false discovery rate (FDR) < 0.05 were identified as a significantly upregulated gene. Both the method used by Li et al. and ours are a standard protocol, and we do not claim which is better because with a threshold of 10 for FPKM, both yielded similar results; the binding motifs of *Pb*AP2-FG2 identified in our study (RGAGAR and its variants) were enriched in upstream region of the significantly upregulated genes with FPKM > 10 detected in the study by Li et al. Therefore, important difference in the methods for RNA-seq analysis was only inclusion/exclusion of genes with low FPKM, which was described in the Results section.

<u>Renaming genes is a tricky business. Is there sufficient data here to fully support this? I'm not sure. I certainly do not think it is a good idea to continue renaming ApiAP2 transcription factors? This will lead to serious confusion in the field. One simple reason is that it is possible that they may serve various functions throughout the entire life</u>

<u>cycle, so will each gene product get a different name every time a new phenotype is published? Also, species-specific differences should be anticipated given differences in the lifecycles. Within each publication it is easy to follow which gene is being discussed, but when comparing across publications it will become increasingly difficult to make comparisons. For example in lines 94, 98, and 100: you call the P. yoelii ortholog "PyAP2-FG2" but Li et al. technically call it "PyAP2-O3". It should be PyAP2-O3 to be in-line with Li et al. and then "PbAP2-FG2" for your publication.</u>

We agree that keeping the consistent name for a protein is preferred to avoid confusion in the field. Meanwhile, we also think that the name for a protein should be derived from its role or function, so that the name could easily lead the readers to imagine/recall the role and function of the protein, especially for those who are new to the study. In the case of AP2-FG2/AP2-O3, the previous name, AP2-O3, which implies its role for ookinete development in line with AP2-O, was given based on the knockout phenotype of its gene. However, the study by Li et al. and our current study revealed that it actually functions as a transcription factor in female gametocytes, not after fertilization, in both *P. yoelii* and *P. berghei*. Therefore, we think that the name, AP2-O3, can mislead readers into a wrong idea and thus should not be kept in the field. To be honest, we wish the name had been changed by Li et al.

As you mention, Li et al. kept the name AP2-O3 for the study in *P. yoelii*, and we admit that changing *Py*AP2-O3 to *Py*AP2-FG2 makes the manuscript confounding. Considering your suggestion, we decided to keep the name for *P. yoelii* (i.e. *Py*AP2-O3) and only changed it for *P. berghei* (*Pb*AP2-FG2).

<u>Part III – Minor Issues: Editorial and Data Presentation Modifications</u>

<u>Reviewer #1: 1- The authors are advised to follow the nomenclature that precedes their work and not to change the genes already named by their colleagues to suit their ideas. Li et al (reference 18) used PyAP2-O3 in their article, which has been changed to PyAP2-FG2 (lines 93-95) in this manuscript, with the authors stating that PyAP2-O3 is the bona fide ortholog of PbAP2-FG2. The reviewer found this very confusing.</u>

We agree that keeping the consistent name for a protein is preferred to avoid confusion in the field. Meanwhile, we also think that the name for a protein should be derived from its role or function, so that the name could easily lead the readers to imagine/recall the role and function of the protein, especially for those who are new to the study. In the case of

AP2-FG2/AP2-O3, the previous name, AP2-O3, which implies its role for ookinete development in line with AP2-O, was given based on the knockout phenotype of its gene. However, the study by Li et al. and our current study revealed that it actually functions in female gametocytes, not after fertilization, in both *P. yoelii* and *P. berghei*. Therefore, we think that the name, AP2-O3, can mislead readers into a wrong idea and thus should not be kept in the field. To be honest, we wish the name had been changed by Li et al.

As you mention, Li et al. kept the name AP2-O3 for the study in *P. yoelii*, and we admit that changing *Py*AP2-O3 to *Py*AP2-FG2 makes the manuscript confounding. Considering that and the suggestion also made by reviewer #3, we decided to keep the name for *P. yoelii* (i.e. *Py*AP2-O3) and only changed it for *P. berghei* (*Pb*AP2-FG2).

2- The authors compared their data with those of Li et al (reference 18). They reanalyzed all omic data, starting with the raw data deposited by Li et al, and reinterpreted their data in a very parsimonious way, concluding that they were wrong, and finally proposing their model as the true model. In my opinion, it would be beneficial if the authors were less direct in their criticisms (perhaps they should curb their enthusiasm in "killing" a competing story) and leave room for alternative hypotheses. I suggest rewriting this part with less "enthusiasm," I would say.

Thank you for your opinion. We performed a fairly standard analysis using their data, and our reanalysis was enough to prove that the binding motif they predicted was not the *cis*-regulatory repressive element that associates with *Py*AP2-O3 and that *Py*AP2-O3 does not globally repress male genes. We think there is no room to leave for their hypotheses.

3- Missing scales in Figures :
• y-axis scale in figures 1B, 7B
• bar scale in figuress 1C-E

We added those missing scales. Thank you for the notice.

4- Figure 1D : The parental strain must be shown in parallel to demonstrate that there is no difference in the morphology of gametocytes produced by a mutant pbap2-fg2(-) background.

Thank you for the notice. We added representative images of WT gametocytes in Fig 1D (Fig 1C in the revised manuscript).

5- ChIPseq figures 3A and 6A : Rather than plotting a genomic area, the authors should compare enrichment levels across the genome by plotting pairwise correlations between replicates (i.e., plotting the average tag number of rep_1 versus rep_2 enrichment).

Thank you for your suggestion. As you mention, the figures only show a part of the genome. However, we prefer to use peak images to directly show how similar individual peaks look even if the images show only a part of the genome. For the genome-wide comparison, we presented IDR1D analysis in Fig 3B and heatmap in Fig 6D (Fig 6B in the previous manuscript).

Reviewer #2: 1. The authors pursue the characterization of PBANKA_1015500, which contains both an ApiAP2 and an ACDC domain. This protein has previously been characterized by Li et al. 2021 (PMID: 33665945) as AP2-O3. Here the authors chose to re-name this protein to AP2-FG2. Little has been done to prove this protein has no role in male gametocytes or other stages of development. Since there is no official naming convention, anyone can name them based on the role of these proteins in various stages and species. Have the authors considered not renaming the protein for consistency in the field?

We agree that keeping the consistent name for a protein is preferred to avoid confusion in the field. Meanwhile, we also think that the name for a protein should be derived from its role or function, so that the name could easily lead the readers to imagine/recall the role and function of the protein, especially for those who are new to the study. In the case of AP2-FG2/AP2-O3, the previous name, AP2-O3, which implies its role for ookinete development in line with AP2-O, was given based on the knockout phenotype of its gene. However, the study by Li et al. and our current study revealed that it actually functions in female gametocytes, not after fertilization, in both *P. yoelii* and *P. berghei*. Therefore, we think that the name, AP2-O3, can mislead readers into a wrong idea and hence should not be kept in the field. To be honest, we wish the name had been changed by Li et al.

As you mention, Li et al. kept the name AP2-O3 for the study in *P. yoelii*. Considering that and the suggestion made by reviewer #3, we decided to keep the name for *P. yoelii* (i.e. *Py*AP2-O3) and only changed it for *P. berghei* (*Pb*AP2-FG2).

2. In Figure 1B, the IFAs used to substantiate the lifecycle stage-specific expression of AP2-FG2 are very difficult to discern. However, the quality of the image makes it very

hard to properly assess this claim. The authors should support this assertion via an orthogonal method, such as Western Blot analysis at every stage of development. In addition, the authors should quantify their claim of stage-specific expression. Please quantify the percentage of males and females that have GFP expression.

We claim that a nuclear-localized GFP signal was clearly shown for female gametocyte in Fig 1B. Meanwhile, as described above, no obvious fluorescence was detected in any other stages of the *Pb*AP2-FG2::GFP, especially in their nucleus. We think that other methods, such as Western blot analysis will not change such a clear, consistent result and hence are not required. Also, quantifying the percentage of males and females that have GFP expression is not informative since all females express GFP-fused *Pb*AP2-FG2 while no male does. *Pb*AP2-FG2 is not a protein that is expressed in a mutually exclusive manner.

3. The authors pursue the characterization of PBANKA_1015500, which contains both an ApiAP2 and an ACDC domain. This protein has previously been characterized by Li et al. 2021 (PMID: 33665945) as AP2-O3. Here the authors chose to re-name this protein to AP2-FG2. Little has been done to prove this protein has no role in male gametocytes or other stages of development. Since there is no official naming convention, anyone can name them based on the role of these proteins in various stages and species. Have the authors considered not renaming the protein for consistency in the field?

<Same as the first comment.>

4. In line 128-129 the authors state two clonal lines were produced but never clarify which of the clones were used in each experiment, with the exception of their mosquito feeding experiments in line 135-136.

Thank you for your notice. We performed phenotype analyses of *pbap2-fg2*(-) for both clones except for the cross-fertilization assay, which was only performed with the clone 1. We realize that our statement does not describe this information as you mention. Therefore, we made changes in the manuscript to clearly indicate which clones were used for each experiment.

5. Are the images in Figure 1D and E representative images? Did the authors quantify

morphologically normal/abnormal gametocytes and ookinetes compared to the WT parent? This developmental phenotype should be quantified for each knockout clone. Additionally, the WT representative images should be shown for comparison (Figure 1D).

Thank you for your comment. Yes, the images in Fig 1D (Fig 1C in the revised manuscript) and 1E are representative images. To make it clear, we added the word "representative" in the Figure captions. In addition, we added the representative images of WT gametocytes for Fig 1D.

About quantification, we have roughly counted the number of normal/abnormal cells for each sexual-stage but omitted the data because it was basically the same as the results previously reported by Modrzynska *et al* (2017) and thus seemed redundant to be mentioned. For the same reason, we omitted the data about exflagellation and oocyst number, which you mentioned in the following comments #6 and #7. However, we realize that it is important to thoroughly demonstrate consistency between our results and previous results, since our mutant lines are generated independently. Therefore, we properly quantified normal/abnormal cells and presented in Fig 1C and 1E.

6. In line 131-132 the authors assert that the ap2-fg2 knock out line produces normal amounts of exflagellated parasites but do not provide any supporting data. Please quantify the number of exflagellation events for both clones.

We quantified the number of exflagellation centers at 12 min after induction of gamete activation for WT and two clonal lines of *pbap2-fg2*(-). The experiment was performed in a gametocyte-enriched condition, i.e. infected mice were treated with sulfadiazine in their drinking water to kill asexual parasites. We added the graph as Fig 1D.

7. Line 135-136 the authors also do not show data to support the assertion that there is a loss of ookinete production in the ap2-fg2 knock out line (either clone).

For confirmation, since you referred to line 135-136, we assumed that you meant "a loss of oocyst" instead of "a loss of ookinete". We depicted the graph showing the number of oocysts at 14 dpi for WT and two clonal lines of *pbap2-fg2*(-). We added the graph as Fig 1F.

8. Figure 1F is missing from the manuscript file, the authors should also state how

many biological and technical replicates were performed for the experiments whose data is presented in these panels.

Thank you for your kind notice. We are sincerely sorry that we misplaced Fig 1F in the Figures. We added the appropriate graph as Fig 1G due to the removal of Fig 1B and addition of two figures mentioned above. Number of replicates was three. We described it as “Error bars indicate the standard error of the mean (n = 3).” in the figure caption.

9. The authors should clarify the remark, “overall, log2(fold change) of female-enriched genes tended to be lower than the other genes” (line 174-176). Is this in context of the significantly decreased transcripts only or the total transcriptome? Comparing the expression of these classes of genes with a two-tailed t-test is also not strictly appropriate, as there would be thousands of genes falling into the other category that are not significantly different between WT and KO. Perhaps the more appropriate test would be to perform a Fisher’s exact test to check for relative overrepresentation of female-enriched genes in the significantly decreased transcripts identified in the study.

We meant the total transcriptome; hence, approximately 500 female genes vs 4000 other genes. To make the statement clear, we rephrased the “overall” to “in the total transcriptome”.

We understand your concern about using log2(fold change) values for genes whose expression was not significantly different between WT and *pbap2-fg2*(-) in the statistical analysis. However, each log2(fold change) values were calculated using three independent data for each sample, so the values themselves have certainty whether they are significant in the aspect of difference between WT and *pbap2-fg2*(-) or not. In addition, it is not likely that significant difference in distribution of log2(fold change) values between two groups of genes, with p-values of 1.4×10^{-18}, was just detected by chance. Therefore, we think that the two-tailed Student’s t-test could be valid.

About the Fisher’s exact test, we found your suggestion very helpful. Thus, we performed the test and described the result in Line 182-183.

10. The authors determined the genomic location of their called peaks from the ChIP-seq. Out of the almost 3,000 peaks from both experiments, what was the distribution of the peaks in intergenic vs intragenic regions? For those that did not have a recognized motif, was the distribution different?

We investigated the location of peaks and found that 1127/1321 common peaks and 558/638 highly reproducible peaks located in intergenic regions. We described the result in the Results section as follows;
Line 229-230: "The analysis revealed that of the 638 highly reproducible peaks, 558 peaks located in intergenic regions".

Between the distribution of peaks with and without motifs, there was no obvious difference; of the 540 highly reproducible peaks that have motif within 300 bp from the summit, 467 peaks located in intergenic regions.

11. The authors postulate the role of AP2-FG2 as a transcriptional repressor and the authors note in Table S3 whether or not each potential target gene is significantly up or down regulated in the AP2-FG2(-) transcriptomics. To assess the significance, the authors should present the distribution (not necessarily significantly up or down) of all the identified ChIP target genes in the transcriptomics experiments.

Thank you for your comment. As we have stated in Line 269-272, overall the target genes of *Pb*AP2-FG2 were upregulated in *pbap2-fg2*(-); "Furthermore, comparing the $\log_2$(fold change) distribution of the target genes with that of the other genes revealed that the target genes tended to be upregulated in *pbap2-fg2*(-) with a *p*-value of 8.2×10^{-58} by two-tailed Student's t-test." In addition, the distribution of all target genes in the differential expression analysis was depicted in the volcano plot (Fig 4A). To make the result clearer to readers, we added a column for "log2(Fold Change) [*pbap2-fg2*(-) vs WT]" in S3C Table.

12. Line 308-311: To demonstrate sex-specificity using the RT-qPCR, the authors failed to use a published Why did the authors use a male and female marker to compare the RT-qPCR results to instead of a published reference gene?

Thank you for your question. We suspected that the RT-qPCR result might be affected by the difference in ratio of early, male and female gametocytes in the samples, and hence, each sex-specific gene would be a better control to calculate the relative amount of *psh3* transcripts.

13. The authors also assert in their heading line 315-316 that AP2-FG2 requires a corepressor to repress its target genes, but the authors do not show that the binding of

AP2-FG2 is lost in the absence of AP2R-2.

Thank you for your comment. As you suggest, we have not confirmed whether *Pb*AP2-FG2 binds to the genome in the absence of *Pb*AP2R-2. However, in the heading, we meant that *Pb*AP2-FG2 activity as a repressor is lost in the absence of *Pb*AP2R-2, whether or not it binds to the genome. This conclusion was derived from the highly similar phenotype of *pbap2-fg2*(-) and *pbap2r-2*(-) in the transcriptomic level. Moreover, thanks to all three reviewers, the additional RIME experiment proved formation of complexes between *Pb*AP2-FG2 and *Pb*AP2R-2 and further supported the conclusion.

14. The authors claim that the genome-wide distribution of AP2-FG2 and AP2R-2 are highly similar based on data shown in Figure 6A and B (Lines 329-330). However, Figure 6A shows only a small portion of the genome and Figure 6B shows read coverage data instead of log2 fold enrichment. It would be more convincing if the authors instead showed the overlapping peaks the way they show for the overlap with AP2-G (figure 7A) or alternatively use the IDR1D analysis.

Thank you for your suggestion. We added a Venn diagram showing an overlap of peaks between ChIP-seq of *Pb*AP2-FG2 and *Pb*AP2R-2 in Figure 6. For Fig 6B (Fig 6D in the revised manuscript), the result would not be significantly different if log2 fold enrichment over an input data was shown (the heatmaps showing log2 fold enrichment are presented below). Therefore, we think the heatmaps in Fig 6D very well presents the high similarity of peak distribution between the two experiments.

We understand that ChIP-seq data are often shown as log2 fold enrichment in *Plasmodium* studies. Meanwhile, considerable number of studies for mammals and yeast etc show ChIP-seq data as read coverage. We prefer showing read coverage of IP data because it directly shows the level of background fragments in the IP samples, hence quality of the IP experiments. In the case of showing ChIP-seq data as log2 fold enrichment, normalization by the input visually lowers the background levels, and quality of the IP experiments cannot be directly evaluated.

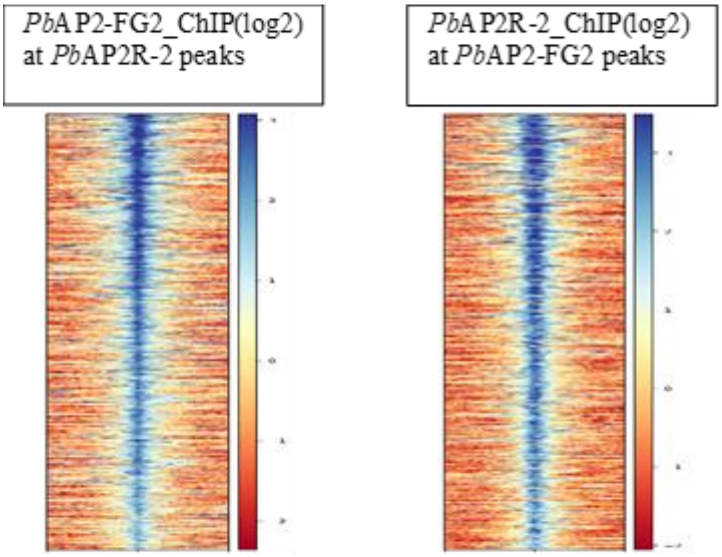

15. The way the authors showed the overlap between the increased and decreased transcripts between AP2-FG2 and AP2R-2 in Figure 6E with a correlation plot is not appropriate for the distribution of the data. For a linear correlation in the data to be drawn, the data needs to follow a linear distribution, which is not the case for their increased and decreased transcripts. The authors can simply show what proportion of transcripts are differentially abundant in each of the conditions.

Thank you for your suggestion. Since we proved that *Pb*AP2-FG2 and *Pb*AP2R-2 form a complex by the RIME (thanks to all three reviewers' suggestions), we think that the data showing linear correlation between log2(fold change) of *pbap2-fg2*(-) vs WT and *pbap2r-2*(-) vs WT, which was only an indirect proof for association of *Pb*AP2-FG2 and *Pb*AP2R-2, can be omitted. However, we are not sure how the analysis was not appropriate, reading your statement that "For a linear correlation in the data to be drawn, the data needs to follow a linear distribution, which is not the case for their increased and decreased transcripts." We performed linear approximation (actually linear regression) to show how much the data can fit to a linear equation with R-squared. If necessary, we could provide *t*-value and *p*-value for a slope of the regression equation, which were 30 and 1.8×10^{-90}, respectively. We would really appreciate if you could teach us textbooks or studies that explain your statement in detail, and that will be considerably helpful for us to improve our future studies.

16. Please scale ChIP-seq tracks between groups and include y- and x-axes on ChIP-seq tracks for figures 1,3,6 and 7. When showing ChIP-seq tracks also clarify whether

what is being displayed is truly "coverage" or actually log2(ChIP/input).

Sorry for the lack of information in the figures showing ChIP-seq tracks. We described y- and x-axis scales in the figures you mention. We also described that the read coverage for ChIP data was shown in these figures in the Figure captions.

17. In Supplementary Figure 1: the authors should state which primer sets illustrated in the pictograms produced the PCR products shown in the figure and the conditions of the PCR should be stated: nr of cycles, annealing temperature of primers should be included in supplementary table S8.

Thank you for your suggestion. We modified S1 Fig (S2 Fig in the revised manuscript) accordingly and described conditions of the PCR in the Materials and Methods as follows; Line 705-707. "The PCRs were performed using PrimeSTAR GXL DNA Polymerase (Takara). PCR products were amplified for 30 cycles, and annealing temperature was set at 55 °C."

Since we employed the same conditions for all PCR experiments, including those for donor construction and genotyping, we indicated this information on the top of S8 table instead of stating for each primer.

18. Line 455-457: The authors state they set their FPKM threshold at <10, but it seems they meant to include transcripts with FPKM >10.

Thank you for your comment. As you suggest, we meant that we have included transcripts with FPKM > 10 in the analysis. We removed ">" to make the statement correct.

19. Please revise or complete the following requests for the Materials and Methods section of the paper:
a. Please include a detailed methodfor the "fluorescence analysis" of the parasites-including what microscope was used, if this was an immunofluorescence assay or live fluorescence microscopy etc.

We added the "fluorescence analysis" method in the Materials and Method section as follows;
Line 710-713. "Expression of GFP-fused *Pb*AP2-FG2 was analyzed by live fluorescence microscopy. Cells were stained with 1 ng/mL Hoechst 33342 for 10 min. Images were

taken by Olympus BX51 microscope with Olympus DP74 camera.”

b. For the generation of plasmid for transgenic alteration of the parasite, include more details on the plasmid background, with appropriate references, should be stated clearly, with a higher level of detail than simply naming them: ie. “gfp-fusion vector” and “targeting construct”

We are sorry that description of plasmid constructs was insufficient in the manuscript. We rewrote the methods as follows and added a schematic illustration of *gfp*-fusion vector in S2A Fig;

Line 680-691. “Briefly, for *gfp*-tagging, two homologous regions were cloned into the *gfp*-fusion vector to fuse *pbap2-fg2* in-frame with *gfp*. The *gfp*-fusion vector was previously generated on the backbone of pBluescript SK (+) (Stratagene, La Jolla, CA, USA). In this vector, 3' untranslated region (UTR) of *hsp70* and a *hdhfr* expression cassette (under the control of *pbef-1α* promoter and 3' UTR of *pbdhfr*) were located 3'-side of the *gfp* gene in this order. Of the two homologous regions, one was cloned to 5'-side of the *gfp* using *Xho*I/*Nhe*I site, and the other was cloned to 3'-side of the *hdhfr* cassette using *Bam*HI/*Not*I site (S2A Fig). The plasmid was linearized by *Xho*I and *Not*I digestion before use in transfection experiments. To knock out *pbap2-fg2*, the targeting construct was prepared using overlap PCR. The construct had two homologous regions around the *pbap2-fg2* locus flanking a *hdhfr* expression cassette, which was derived from the *gfp*-fusion vector described above.”

c. The authors stated the parasite lines were verified by PCR and/or Sanger sequencing. Please specifically state which methods were used in which cases, if Sanger sequencing was used to verify these lines please provide supplementary data to this effect.

Sorry for our unclear statement. We only performed Sanger sequencing for the motif_mutated parasite, the result of which was provided in Fig 5A. To clarify, we made a change in the sentence as follows;

Line 704-705. “Recombination was confirmed by PCR and, for the motif_mutated parasite, Sanger sequencing.”

d. The authors should consider performing next-generation whole genome sequencing on the knockout parasite lines to verify that there are no other mutations that could contribute to the phenotype.

Thank you for your comment. We think that performing NGS of the whole genome every time someone develops a knockout line seems too much work for a phenotype analysis. To exclude possibility of existence of other mutations that contribute to a certain phenotype, we assess two independent clones for each knockout experiment.

e. For the ChIP-seq experiments- there seems to be substantial information about the experimental protocol missing without any supplementary method being cited. Please provide a more detailed protocol and ensure to include the following detail OR cite the source of the protocol that includes these details: Was a nuclear extraction performed prior to sonication? What is the composition of the SDS lysis buffer? At which temperature were the samples sonicated? What was the concentration of anti-GFP antibodies used in the ChIP-seq experiment and how long was the antibody incubation performed? Were the lysates precleared before the immunoprecipitation step? When were the input control samples taken? The authors also don't mention a proteinase digestion step, was this omitted? How was the immunoprecipitated DNA purified?

We are sorry for the lack of information in our ChIP-seq method. We rewrote the method as follows;
Line 735-753. "Whole blood was withdrawn from the infected mice treated with sulfadiazine and passed through the Plasmodipur filter to remove white blood cells. The blood was diluted in a complete medium (RPMI1640 supplemented with 20% fetal calf serum) and immediately fixed with 1% formalin at 30 °C. After fixing, RBCs were lysed in ice-cold 1.5 M NH4Cl solution. This step was performed several times until the supernatant became clear, and the cells were lysed in SDS lysis buffer (50 mM Tris-HCl, 1% SDS, 10 mM EDTA). The samples were sonicated at 4 °C using Bioruptor (Cosmo Bio) for 20 cycles of 30 sec on/30 sec off to shear the chromatin. Input samples were collected at this point from the sonicated cell lysate. For IP samples, chromatins were immunoprecipitated with anti-GFP polyclonal antibodies (5 mg/mL; Abcam, ab290), which were bound to Protein A Magnetic Beads (Invitrogen) before the ChIP step (2 µL of antibody was mixed with 20 µL of beads and incubated for > 1 h). After 12 h of incubation at 4 °C, the beads were washed with low-salt buffer (20mM Tris-HCl, 0.1% SDS, 1% Triton X-100, 2 mM EDTA, 150 mM NaCl) for five times and high-salt buffer (20mM Tris-HCl, 0.1% SDS, 1% Triton X-100, 2 mM EDTA, 500 mM NaCl) for three times. Immunoprecipitated chromatin was then eluted in elution buffer (10mM Tris-HCl, 1% SDS, 5 mM EDTA, 300 mM NaCl), heated at 65 °C for 8 h, and processed with RNase

H for 1 h and proteinase K for 2 h. DNA fragments were purified from the immunoprecipitated chromatin by phenol/chloroform extraction and ethanol precipitation and used for library construction."

To clarify, we did not perform nuclear extraction or preclearance of cell lysate with antibody-free protein A beads.

f. There is also information missing regarding the data analysis of the ChIP-seq experiments: What version of the Plasmodium berghei genome was used to map the sequencing reads? How/why did the authors decide on a fold change enrichment threshold of >3?

The genome sequence data was version 3 obtained from PlasmoDB 46. We added this information in the Materials and Methods. Thank you for the notice.

About decision of a fold enrichment threshold for ChIP-seq analyses, most studies set the threshold between 2 and 4 without providing any particular reason why the value was selected, to the best of our knowledge. Thus, we think setting the fold enrichment threshold as 3 is fairly standard. In the present manuscript, we preferred the value for several reasons, including overlap of peaks in duplicates and ratio of peaks with the enriched motifs. Just to be clear, we used the criteria provided in the *P. yoelii* study, in which a fold enrichment threshold was 2, to reanalyze the ChIP-seq data of *Py*AP2-O3 because we thought that would be fair to the previous study.

g. For the ChIP-qPCR and RT-qPCR experiments, what were the starting concentrations of DNA or RNA included in the experiments respectively and how many cycles were these experiments conducted for? Did the authors include reference genes for either study for normalization? No account is given of how these data were analyzed, was the 2-delta Ct method used for quantification in the qPCR?

For the ChIP-qPCR analysis, we did not quantify the concentration of DNA samples. Since IP and input samples were harvested from 200 μL and 10 μL of the cell lysate, respectively, the %input values were simply calculated as $2^{\wedge}(Ct^{IP}\text{-}Ct^{input}) \times (1/20) \times 100$ without normalization by reference genes. For the RT-qPCR, cDNA was synthesized from 1 μg of total RNA, and 1/200 of the synthesized cDNA was used for qPCR. Amplification step of qPCR was conducted for 40 times, and Ct values ranged from 20 to 35. We described these details as follows;

Line 774-785. "IP and input samples were each prepared from 200 μL and 10 μL of

sonicated cell lysate, respectively. Quantification of DNA fragments of interest was performed by real-time qPCR using TB Green Fast qPCR Mix (Takara) and Thermal Cycler Dice Real Time System II (Takara). Amplification cycles were performed for 40 cycles, and cycle threshold (Ct) was detected between 20 to 35 cycles. From the Ct values, %input values were calculated as $2^\wedge(Ct^{IP}\text{-}Ct^{input}) \times (1/20) \times 100$. Three biologically independent experiments were performed and used for the analysis.

For RT-qPCR analyses, cDNA was synthesized from 1 μg of total RNA, extracted as described for RNA-seq analysis, using the PrimeScript RT reagent Kit with gDNA Eraser (Takara), and 1/200 of the synthesized cDNA was used for qPCR. Real-time qPCR experiments were performed as described above.”

20. Why did the authors use the Fisher’s exact test to analyze enriched motifs in 6 bp bins instead of using available softwares (ie STREME) that scan over a wider range? Could you please justify your use of the Fisher’s exact test in this context?

We perform enrichment analysis using the Fisher’s exact test on all motifs, so that all candidates of possible binding motifs can be detected. In addition, because enrichment of a motif in peak regions is analyzed against the other genomic regions, sequence bias in the genome can be considered in this analysis. In the present study, we only showed enrichment analysis of 6-bp motifs, but usually, we perform the analysis on motifs with various length, mostly from 5-bp to 10-bp. We chose the length considering their p-values and how many peaks contain the motif within the peak region.

We agree that currently available software, such as MEME and STREME, is also useful. However, sometimes these methods produce incorrect binding motifs, which has been seen in the Py study, for some reasons. We suppose that how you prepare control sequences somehow affects the result. We think that our analysis is more thorough and simpler and hence able to discover a true binding motif although the unification of enriched motifs needs to be done manually.

21. How did the authors associate genes with Chip-seq peaks if the binding site overlaps within 1200 bp of the start codon? This method does not take into account which genes are closest to the binding site or whether these bound regions are intra- or intergenic.

In our analysis, we actually measured distance between peak summit and a start codon for each gene. However, we decided to include both genes in the targets if a peak located within 1200 bp of two genes because bidirectional promoters are often observed in the

Plasmodium genome, especially for small intergenic regions. In addition, because the distance between TSS and start codon varies depending on genes, not always the closer gene is the true target for a binding site. To include as many true targets as possible, we do not consider the distance a peak summit and a start codon for target analysis.

Targets were predicted from peaks on intergenic regions, and any peak on intragenic regions was not used for target prediction. The "within 1200 bp" threshold is only set for genes whose upstream intergenic region was larger than 1200 bp. To clarify this method in the manuscript, we made a change in the Results section as follows;
Line 229-232. "The analysis revealed that of the 638 highly reproducible peaks, 558 peaks located in intergenic region. These intergenic peaks located in the upstream regions (within 1200 bp from ATG) of 517 genes, suggesting that these genes are a target gene of *Pb*AP2-FG2 (S3C Table)."

Reviewer #3: In general, please check the entire document for English grammar. There are examples beginning with the second sentence of the Abstract that require further attention.

Thank you for your notice. We have thoroughly checked the manuscript and corrected grammatical errors, including the one in the second sentence of the Abstract, as many as we could find.

The PlasmoDB IDs for all genes mentioned in the manuscript should be provided, especially since the authors are choosing to rename pbap2-o3 as pbap2-fg2. (Such as line 317 and others throughout.)

Thank you for your suggestion. We added the PlasmoDB IDs for all genes at their first appearance in the Results section of the manuscript.

The word "identical" is used throughout the manuscript. All instances should be replaced with "highly similar" or "analogous", etc… In biology virtually nothing is identical.

Thank you for the advice. We realize that the word "identical" was not appropriately used in our manuscript. We rephrased the word "identical" throughout the manuscript according to your comment.

Line 68 – Probably want to use "ability" instead of "capability".

Thank you for the correction. We rephrased accordingly.

Please be consistent in the use of AP2R-2 instead of ap2-R2 (see lines 84 and 86). Please correct throughout the manuscript.

Thank you for your kind notice. We are sorry for being careless. We corrected the mistakes properly.

The last section of the Introduction (lines 96-100) should be rewritten to simply state that a re-analysis of a previous PyAP2-FG2 dataset leads to similar conclusions despite being at odds with what was previously reported in the Py study.

Thank you for your suggestion. We rewrote the last section as follows;
Line 104-106. "Although some of their conclusions were inconsistent with those in this study, reassessment of their data led to similar conclusions in both *P. berghei* and *P. yoelii*."

Lone 110-111, it is unnecessary to state what a previous study failed to do. Alternatively, they may suggest that a gene may have an earlier role leading up to the point of the observed phenotype used to name ap2 genes earlier (by Modrzynska and others). This is especially true when in lines 135-137 the authors compare their results in a confirmatory manner with this previous study.

We realize that our statement seemed to demean the previous study as you suggest. Considering your comment into account, we rewrote the sentence as follows;
Line 116-118. "This result indicated that *Pb*AP2-FG2 has a role in zygote/ookinete development or regulating female transcriptome because female gametocytes store large amount of mRNAs that are essential for development after fertilization."

On line 145, please clarify why 30% conversion (and not 50%) to banana-shaped ookinetes is expected?

First, we are sincerely sorry that we misplaced Fig 1F (Fig 1G in the revised manuscript) in the manuscript. As you suggest, theoretically, the conversion rate should be 50%, but it is actually difficult to reach the theoretical value in a cross-fertilization assay. In fact,

many studies report 30-40% conversion in cross-fertilization assays, or sometimes even less than 30%. In addition, even when WT is cultured, the conversion rate will be 80-90%. In our current study, the conversion rate was also less than 40% when *p48/45*(-) and *p47*(-) were crossed (Fig 1G). Therefore, we concluded that male gametocytes of *pbap2-fg2*(-) were normal.

Please clarify the sentence on lines 224-226: "The target genes contained some groups of genes seemingly expressed in female gametocytes…" What does it mean to be "seemingly expressed"?

Sorry for the unclear statement. We rewrote the sentence as follows;
Line 235-237. "The target genes contained some groups that included many genes highly transcribed in female gametocytes, such as "cytoskeleton" and "secretory protein"."

Again, on lines 244-234, the authors write: "…the number of male-enriched genes in the targets seemed too large, again implying the possible role of PbAP2-FG2 as a transcriptional repressor." You cannot write "seemed too large" unless you have a comparative analysis? On what basis do the authors make this claim?

We realized that the statement was ambiguous and irresponsible as you mention. Thank you for the indication. We rewrote the sentence as follows;
Line 254-256. "Because *Pb*AP2-FG2 is a female-specific transcription factor, the considerable number of male-enriched genes in the targets may imply the possible role of *Pb*AP2-FG2 as a transcriptional repressor."

In lines 248-249, the authors should note that it is very difficult to distinguish the direct vs. indirect targets by RNA-seq analysis alone, therefore target genes need to be identified within the DEGs.

Thank you for the suggestion. That is a very important point that we would like to indicate in our manuscript. Taking your advice, we added the following sentence in the Results section;
Line 286-290. "In addition, this comparison of the differential expression analysis and target analysis indicated the importance of target analysis by ChIP-seq for identifying direct targets of a transcriptional regulator because it is difficult to distinguish direct and indirect effects resulted from a gene knockout by RNA-seq analysis alone."

The candidate gene psh3 used the section beginning on lines 277-278 was selected due to its expression and essentiality in P. falciparum parasites. Is this essentiality also true in P. berghei (PlasmoGEM or other datasets?). Also, doesn't this imply that any regulation via PbAP2-FG2 must be exclusive to the sexual stage and that the expression in trophozoites and schizonts must be via a different factor in the asexual blood stages? I suppose that I'm not convinced as to why psh3 was used for this validation? Why not use a gene that has been well-characterized in Pb?

As in *P. falciparum*, *psh3* is also suggested to be essential in *P. berghei* according to the PlasmoGEM. We described this in the Results section as follows;
Line 302-303. "In addition, *psh3* is also reported to be essential for asexual development of *P. berghei* in the PlasmoGEM study."

About transcriptional regulation of *psh3*, *Pb*AP2-FG2 is not involved in the expression of *psh3* in trophozoites and schizonts because it is a repressor in female gametocytes. What we attempted to investigate in the reporter assay was repression of *psh3* transcription in female gametocytes by *Pb*AP2-FG2 on its binding motif. About activation of *psh3* during asexual blood stages, we agree with your suggestion that different transcription factors are involved in the process, probably with a different *cis*-regulatory element.

The reason why we selected *psh3* as a target for the reporter assay was that besides its low expression in female gametocytes, its upregulation in *pbap2-fg2*(-) and *pbap2r-2*(-) was both significant in the differential expression analysis. These results made us confident that psh3 is a true target of the *Pb*AP2-FG2-*Pb*AP2R-2 complex. To clarify this, we added the following sentence in the Results section;
Line 305-307. "In the differential expression analysis between WT and *pbap2-fg2*(-), *psh3* was significantly upregulated in *pbap2-fg2*(-) with $\log_2$(fold change) of 2.0, assuring that it is a true target gene of *Pb*AP2-FG2."

Line 317, please spell "Previously" correctly.

Thank you for the notice. We corrected the spell.

Line 348, delete "were".

Thank you for the correction. We deleted the "were".

Line 362, "approximately 1" should be 0.97.

We changed the phrase according to your comment.

Line 365-366 should read: "Therefore we hypothesized that AP2R-2 may function as an essential co-repressor of PbAP2-FG2 in female gametocytes."

We changed the sentence accordingly.

Lines 371-372 are unclear. What "changes" is not well defined.

We are sorry for the ambiguous statement. We changed the sentence as follows;
Line 441-442. "This result indicates that a major transcriptional activator switches from *Pb*AP2-G to *Pb*AP2-FG as early gametocytes develop into females."

In lines 376-378 it is reported that the targets of AP2-G (Pb I presume?) and PbAP2-FG2 overlap, but what about the mRNA abundance in the knockouts. Does the abundance go up (activated, or loss of repression) after PbAP2-FG2 knockout? Please clarify.

Thank you for the insightful suggestion. We did not think of investigating upregulation of the common targets, which was very important for our conclusion, since the target genes were overall upregulated. We evaluated the upregulation of the common target genes and described the result as follows;
Line 449-452. "In addition, the distribution of $\log_2$(fold change) values for these common target genes in the differential expression analysis between WT and *pbap2-fg2*(-) was higher than that for the non-target genes with p-value = 1.4×10^{-13} by two-tailed Student's t-test, suggesting that these genes are actually repressed in female gametocytes."

Line 414, do not use "implausible". Please replace with "unlikely".

We replaced the "implausible" with "unlikely".

In line 435, how is it possible that only 271 of 781 targets genes have orthologues between Py and Pb? These organisms are remarkably similar overall. Please clarify.

Thank you for your comment. As you expect, most of the target genes have orthologues between *Py* and *Pb*. We meant that 271/781 target genes were also found in the targets of *Pb*AP2-FG2 as an orthologous gene. We are sorry for the inaccurate sentence. We changed the sentence as follows;
Line 511-512. "The analysis identified 781 target genes, 271 of which were included in the target genes of *Pb*AP2-FG2 as an orthologous gene"

<u>On line 440, you state that "…some targets might have been falsely detected." It is not clear how a target can be falsely detected. Surely based on the analysis used by Li et al. they did measure a target peak.</u>

Thank you for the comment. As you suggest, the target analysis by Li et al. was valid, and our statement was not appropriate. What we wanted to mention was that due to the low reproducibility of their ChIP-seq peaks, some targets could be derived from unreliable peaks and might not be a true target. Therefore, we changed the sentence as follows;
Line 515-517. "Given the low reproducibility of the ChIP-seq data, we supposed that some targets might have been derived from unreliable peaks and hence not a true target."

<u>In lines 440-441, how did you "…assess the sex-specific expression of the target genes of PyAP2-FG2…"?</u>

We defined orthologous genes of *Pb* female- and male-enriched genes in *P. yoelii* as *Py* female- and male-enriched genes, respectively. To clarify that in the manuscript, we added a sentence in the Results section as follows;
Line 517-519. "We further assessed the sex-enriched expression of the target genes of *Py*AP2-FG2 considering orthologous genes of the female- and male-enriched genes defined above as sex-enriched genes for *P. yoelii*."

<u>You should not write on lines 450-451 "… yield unreliable DEG lists." This is demeaning to the previous study. Perhaps you mean they "… yield much larger DEG lists with larger variance." Or something along those lines?</u>

Thank you for your suggestion. We rewrote the sentence according to your comment.

<u>Line 455 change to "we re-analyzed".</u>

We made the change accordingly. In addition, we changed “we assessed the RNA-seq data” to “we reassessed the RNA-seq data” in Line 525.

How many orthologues were considered in line 461?

In our analysis, 4426 orthologues were considered between *P. berghei* and *P. yoelii* according to the orthology and synteny data in PlasmoDB. We added this information to the Materials and Methods as follows;
Line 730-732. “For comparison between results for *P. berghei* in this study and *P. yoelii* in the previous study, 4426 orthologues were considered according to the orthology and synteny data in PlasmoDB.”

Line 45574 change to “the majority”.

We added “the” accordingly.

Figure comments:
I believe that the Figure 1B ChIP-seq data is published data and should not be in a main figure. Perhaps a supplementary figure?

Thank you for the notice. We removed Fig 1B from the main figures and included it as S1 Fig.

Figure 1: Is the IGV image group auto scaled? Meaning is the y-axis consistent between both tracks? The y-axis ranges should be indicated on the figure or in the caption.

Thank you for the indication. We showed raw read coverage of ChIP data in those figures. Therefore, each group was independently scaled. Sorry for the lack of y-axis scale in the figures. We indicated the y-axis scales in those figures.

Figure 1F: This panel regarding the crossing experiment where you have crossed a female and a male gametocyte infertile lineage with ap2-fg2 (-) is missing. Please add this.

Thank you for your kind notice. We are sincerely sorry that we misplaced Fig 1F in the Figures. We added the appropriate graph as Fig 1G due to the removal of previous Fig 1B and addition of two figures suggested by reviewer #2.

<u>In Figure 2 B and C, are the other genes not assigned to male and female still gametocyte genes? Or genes expressed during other (multiple perhaps?) stages? Please clarify this in the manuscript and figure legend. The same question applies to Figure 3G.</u>

We assigned all genes that are neither female- or male-enriched genes as "other". We realize that this was not clear in the manuscript. Thank you for your notion. We made following changes in the Results and Figure Captions;
Line 179-180. "(all the other genes were assigned as "other" in this context)"
Line 1099-1100. "All genes not enriched in either "female" or "male" are classified as "other"."

<u>In Figure 4, the majority of the differences are really not significant. With a reduced number of candidates, it is not surprising that motif enrichment is stronger. Also, can you explain the data that emanate from the bottom of your plots in the bottom right quadrants in Figure 4A that form a line not congruent with the rest of the volcano plot?</u>

About significance of upregulation of the target genes, we have to admit the limitation in our method for the differential expression analysis. We suppose that if we could perform RNA-seq analyses with female gametocytes sorted by a specific fluorescent marker, like Li et al did in *P. yoelii*, we would have detected more significant upregulation of target genes. For Fisher's exact test, a reduced number of candidates does not always correlate with more significant *p*-values as we understand. In fact, if the ratio of targets in candidates was constant, the significance gets higher as number of candidates increase. In addition, in the case of our motif enrichment analysis for the upstream regions of upregulated genes, sequences were retrieved from 300 to 1200 bp upstream regions of candidate genes (meaning that each region contain 895 6-bp motifs), each of which presumably contains only one or two binding motifs of *Pb*AP2-FG2. Therefore, we believe, if we could detect more upregulated genes, the enrichment of motif would have been stronger.

The right panel of Fig 4A shows a volcano plot magnified from the grid with a red line in the left panel. We thought that too many dots are overlapped in the left panel.

Lines in the right panel are drawn at the same values as the left panel. For Fig 4B and 4C, we set the vertical line at 0 because we focused on distribution of the target genes rather than significance of their upregulation.

Figure 6B: As you know, it is more widely accepted to show ChIP-seq data as a Log2 fold enrichment of the IP over the input. Why are the data shown as read coverage? From the methods, you demonstrate that you took an input sample, so it would be a nice control to show the heatmaps with the input data as the Fold Enrichment (Log2[IP/Input]), instead of just read coverage.

We do not agree with that showing ChIP-seq data as Log2 fold enrichment is a more standard way than showing read coverage. As you would find, considerable number of studies for mammals and yeast etc show ChIP-seq data as read coverage.

We prefer showing read coverage of IP data because it directly shows the level of background fragments in the IP samples, hence quality of the IP experiments. In the case of showing ChIP-seq data as log2 fold enrichment, quality of the IP experiments cannot be directly evaluated. We are aware that several studies in *Plasmodium* performed ChIP-seq experiments with a low background levels, such as those for *Pf*AP2-G and *Pf*HP1. However, we could not have been sure about it until we reassessed their data from the fastq files because normalization by the input could visually lower the background levels. Those quality works should be noticed in the documents and appreciated by the readers, so we show our ChIP-seq data as read coverage, hoping it to be more accepted in the field of *Plasmodium* studies.

In Figure 8A, I would consider switching the 2nd and 3rd columns to make your point more strongly to the reader since the p-value is higher for the GAGA motif.

Thank you for your advice. We modified the figure according to your comment.

In Supplemental Figure S1, please label the genotyping PCR gels more clearly so that it is clear which primers are being used to generate each product. Also it would be helpful to have the parasite line name (i.e., PbAP2-FG2::GFP) above each schematic in this figure. Is the 3`UTR modified in each of these lines?

Thank you for your comment. We are sorry for S1 Fig (S2 Fig in the revised manuscript) being unclear. We modified the figures according to your suggestions.
