# Supplementary material for: PbAP2-FG2 and PbAP2R-2 function together as a transcriptional repressor complex essential for Plasmodium female development
Source: PLoS Pathog. 2023 Feb 13;19(2):e1010890. doi: 10.1371/journal.ppat.1010890 (PMC9956629; doi:10.1371/journal.ppat.1010890)
Supplement: Attachment — Submitted filename: PPATHOGENS-D-22-01649_R1_responses.docx [file ppat.1010890.s015.docx]

***Reviewer #2:***

***1. The authors pursue the characterization of PBANKA_1015500, which***

***contains both an ApiAP2 and an ACDC domain. This protein has previously been characterized by Li et al. 2021 (PMID: 33665945) as AP2-O3. Here the authors chose to re-name this protein to AP2-FG2. Little has been done to prove this protein has no role in male gametocytes or other stages of development. Since there is no official naming convention, anyone can name them based on the role of these proteins in various stages and species. Have the authors considered not renaming the protein for consistency in the field?***

We agree that keeping the consistent name for a protein is preferred to avoid confusion in the field. Meanwhile, we also think that the name for a protein should be derived from its role or function, so that the name could easily lead the readers to imagine/recall the role and function of the protein, especially for those who are new to the study. In the case of AP2-FG2/AP2-O3, the previous name, AP2-O3, which implies its role for ookinete development in line with AP2-O, was given based on the knockout phenotype of its gene. However, the study by Li et al. and our current study revealed that it actually functions in female gametocytes, not after fertilization, in both *P. yoelii* and *P. berghei*. Therefore, we think that the name, AP2-O3, can mislead readers into a wrong idea and hence should not be kept in the field. To be honest, we wish the name had been changed by Li et al.

As you mention, Li et al. kept the name AP2-O3 for the study in *P. yoelii*. Considering that and the suggestion made by reviewer #3, we decided to keep the name for *P. yoelii* (i.e. *Py*AP2-O3) and only changed it for *P. berghei* (*Pb*AP2-FG2).

Thank you for a satisfactory compromise and the above revision.

***2. In Figure 1B, the IFAs used to substantiate the lifecycle stage-specific expression of***

***AP2-FG2 are very difficult to discern. However, the quality of the image makes it very***

***hard to properly assess this claim. The authors should support this assertion via an orthogonal method, such as Western Blot analysis at every stage of development. In addition, the authors should quantify their claim of stage-specific expression. Please quantify the percentage of males and females that have GFP expression.***

We claim that a nuclear-localized GFP signal was clearly shown for female gametocyte in Fig 1B. Meanwhile, as described above, no obvious fluorescence was detected in any other stages of the *Pb*AP2-FG2::GFP, especially in their nucleus. We think that other methods, such as Western blot analysis will not change such a clear, consistent result and hence are not required. Also, quantifying the percentage of males and females that have GFP expression is not informative since all females express GFP-fused *Pb*AP2-FG2 while no male does. *Pb*AP2-FG2 is not a protein that is expressed in a mutually exclusive manner.

While your justification seems reasonable, we do not agree that an absence of information is conclusive in this case. If you are to look closely at your images provided in Figure 1B, there is a faint GFP signal in both the schizont and male gametocyte that is external to the nucleus. Western Blot would verify that any signal being detected is actually full-length tagged protein or background.

In addition, a Western Blot should have been used to validate the tagged line and it’s suitability for ChIP-seq experiments. Prior to use in a ChIP-seq experiment, epitope tagged lines should be validated to express the tagged protein of interest using the same antibody as will be used for ChIP-seq. The protein band should be demonstrated to be within 20% of the size predicted by the coding region. The authors should demonstrate expression (or lack thereof) of the epitope tagged protein by Western Blot at various stages of development and to support the conditions for ChIP-seq.

***3. The authors pursue the characterization of PBANKA_1015500, which contains both an ApiAP2 and an ACDC domain. This protein has previously been characterized by Li et al. 2021 (PMID: 33665945) as AP2-O3. Here the authors chose to re-name this protein to AP2-FG2. Little has been done to prove this protein has no role in male gametocytes or other stages of development. Since there is no official naming convention, anyone can name them based on the role of these proteins in various stages and species. Have the authors considered not renaming the protein for consistency in the field?***

<Same as the first comment.>

***4. In line 128-129 the authors state two clonal lines were produced but never clarify which of the clones were used in each experiment, with the exception of their mosquito feeding experiments in line 135-136.***

Thank you for your notice. We performed phenotype analyses of *pbap2-fg2*(-) for both clones except for the cross-fertilization assay, which was only performed with the clone

1. We realize that our statement does not describe this information as you mention. Therefore, we made changes in the manuscript to clearly indicate which clones were used for each experiment.

Thank you for the above revision.

***5. Are the images in Figure 1D and E representative images? Did the authors quantify morphologically normal/abnormal gametocytes and ookinetes compared to the WT parent? This developmental phenotype should be quantified for each knockout clone. Additionally, the WT representative images should be shown for comparison (Figure 1D).***

Thank you for your comment. Yes, the images in Fig 1D (Fig 1C in the revised manuscript) and 1E are representative images. To make it clear, we added the word “representative” in the Figure captions. In addition, we added the representative images of WT gametocytes for Fig 1D.

About quantification, we have roughly counted the number of normal/abnormal cells for each sexual-stage but omitted the data because it was basically the same as the results previously reported by Modrzynska *et al* (2017) and thus seemed redundant to be mentioned. For the same reason, we omitted the data about exflagellation and oocyst number, which you mentioned in the following comments #6 and #7. However, we realize that it is important to thoroughly demonstrate consistency between our results and previous results, since our mutant lines are generated independently. Therefore, we properly quantified normal/abnormal cells and presented in Fig 1C and 1E.

Thank you for the above revision.

***6. In line 131-132 the authors assert that the ap2-fg2 knock out line produces normal amounts of exflagellated parasites but do not provide any supporting data. Please quantify the number of exflagellation events for both clones.***

We quantified the number of exflagellation centers at 12 min after induction of gamete activation for WT and two clonal lines of *pbap2-fg2*(-). The experiment was performed in a gametocyte-enriched condition, i.e. infected mice were treated with sulfadiazine in their drinking water to kill asexual parasites. We added the graph as Fig 1D.

Thank you for the above revision.

***7. Line 135-136 the authors also do not show data to support the assertion that there is a loss of ookinete production in the ap2-fg2 knock out line (either clone).***

For confirmation, since you referred to line 135-136, we assumed that you meant “a loss of oocyst” instead of “a loss of ookinete”. We depicted the graph showing the number of oocysts at 14 dpi for WT and two clonal lines of *pbap2-fg2*(-). We added the graph as Fig 1F.

Thank you for the above revision.

***8. Figure 1F is missing from the manuscript file, the authors should also state how many biological and technical replicates were performed for the experiments whose data is presented in these panels.***

Thank you for your kind notice. We are sincerely sorry that we misplaced Fig 1F in the Figures. We added the appropriate graph as Fig 1G due to the removal of Fig 1B and addition of two figures mentioned above. Number of replicates was three. We described it as “Error bars indicate the standard error of the mean (n = 3).” in the figure caption.

Thank you for the above revision.

***9. The authors should clarify the remark, “overall, log2(fold change) of female- enriched genes tended to be lower than the other genes” (line 174-176). Is this in context of the significantly decreased transcripts only or the total transcriptome? Comparing the expression of these classes of genes with a two-tailed t-test is also not strictly appropriate, as there would be thousands of genes falling into the other category that are not significantly different between WT and KO. Perhaps the more appropriate test would be to perform a Fisher’s exact test to check for relative overrepresentation of female-enriched genes in the significantly decreased transcripts identified in the study.***

We meant the total transcriptome; hence, approximately 500 female genes vs 4000 other genes. To make the statement clear, we rephrased the “overall” to “in the total transcriptome”.

We understand your concern about using log2(fold change) values for genes whose expression was not significantly different between WT and *pbap2-fg2*(-) in the statistical analysis. However, each log2(fold change) values were calculated using three independent data for each sample, so the values themselves have certainty whether they are significant in the aspect of difference between WT and *pbap2-fg2*(-) or not. In addition, it is not likely that significant difference in distribution of log2(fold change) values between two groups of genes, with *p*-values of 1.4 × 10^-18^, was just detected by chance. Therefore, we think that the two-tailed Student’s t-test could be valid.

About the Fisher’s exact test, we found your suggestion very helpful. Thus, we performed the test and described the result in Line 182-183.

Thank you for the above revision.

***10. The authors determined the genomic location of their called peaks from the ChIP- seq. Out of the almost 3,000 peaks from both experiments, what was the distribution of the peaks in intergenic vs intragenic regions? For those that did not have a recognized motif, was the distribution different?***20

We investigated the location of peaks and found that 1127/1321 common peaks and 558/638 highly reproducible peaks located in intergenic regions. We described the result in the Results section as follows;

Line 229-230: “The analysis revealed that of the 638 highly reproducible peaks, 558 peaks located in intergenic regions”.

Between the distribution of peaks with and without motifs, there was no obvious difference; of the 540 highly reproducible peaks that have motif within 300 bp from the summit, 467 peaks located in intergenic regions.

Thank you for the above revision.

***11. The authors postulate the role of AP2-FG2 as a transcriptional repressor and the authors note in Table S3 whether or not each potential target gene is significantly up or down regulated in the AP2-FG2(-) transcriptomics. To assess the significance, the authors should present the distribution (not necessarily significantly up or down) of all the identified ChIP target genes in the transcriptomics experiments.***

Thank you for your comment. As we have stated in Line 269-272, overall the target genes of *Pb*AP2-FG2 were upregulated in *pbap2-fg2*(-); “Furthermore, comparing the log2(fold change) distribution of the target genes with that of the other genes revealed that the target genes tended to be upregulated in *pbap2-fg2*(-) with a *p*-value of 8.2 × 10^-58^ by two-tailed Student’s t-test.” In addition, the distribution of all target genes in the differential expression analysis was depicted in the volcano plot (Fig 4A). To make the result clearer to readers, we added a column for “log2(Fold Change) [*pbap2-fg2*(-) vs WT]” in S3C Table.

Thank you for the above revision.

***12. Line 308-311: To demonstrate sex-specificity using the RT-qPCR, the authors failed to use a published Why did the authors use a male and female marker to compare the RT-qPCR results to instead of a published reference gene?***

Thank you for your question. We suspected that the RT-qPCR result might be affected by the difference in ratio of early, male and female gametocytes in the samples, and hence, each sex-specific gene would be a better control to calculate the relative amount of *psh3* transcripts.

Thank you for the above clarification. It is unclear to me how your choice is better without seeing the data, but I do believe that you have clearly stated what was done and this is sufficient.

***13. The authors also assert in their heading line 315-316 that AP2-FG2 requires a corepressor to repress its target genes, but the authors do not show that the binding of AP2-FG2 is lost in the absence of AP2R-2.***

Thank you for your comment. As you suggest, we have not confirmed whether *Pb*AP2- FG2 binds to the genome in the absence of *Pb*AP2R-2. However, in the heading, we meant that *Pb*AP2-FG2 activity as a repressor is lost in the absence of *Pb*AP2R-2, whether or not it binds to the genome. This conclusion was derived from the highly similar phenotype of *pbap2-fg2*(-) and *pbap2r-2*(-) in the transcriptomic level. Moreover, thanks to all three reviewers, the additional RIME experiment proved formation of complexes between *Pb*AP2-FG2 and *Pb*AP2R-2 and further supported the conclusion.

We thank the authors for performing the additional RIME experiment that empirically establishes the interaction of PbAP2-FG2 and PbAP2R-2. Could the authors speculate as to why AP2-I, AP2-G2 and AP2-O2 are also found to complex with PbAP2-FG2? This should be added to the discussion of the results of RIME. Additionally, please discuss your results with respect to the recently published *P. berghei* male/female gametoycte differentiation and maturation ssRNA-seq study <https://doi.org/10.1016/j.chom.2022.12.011>.

***14. The authors claim that the genome-wide distribution of AP2-FG2 and AP2R-2 are highly similar based on data shown in Figure 6A and B (Lines 329-330). However, Figure 6A shows only a small portion of the genome and Figure 6B shows read coverage data instead of log2 fold enrichment. It would be more convincing if the authors instead showed the overlapping peaks the way they show for the overlap with AP2-G (figure 7A) or alternatively use the IDR1D analysis.***

Thank you for your suggestion. We added a Venn diagram showing an overlap of peaks between ChIP-seq of *Pb*AP2-FG2 and *Pb*AP2R-2 in Figure 6. For Fig 6B (Fig 6D in the revised manuscript), the result would not be significantly different if log2 fold enrichment over an input data was shown (the heatmaps showing log2 fold enrichment are presented below). Therefore, we think the heatmaps in Fig 6D very well presents the high similarity of peak distribution between the two experiments.

We understand that ChIP-seq data are often shown as log2 fold enrichment in *Plasmodium* studies. Meanwhile, considerable number of studies for mammals and yeast etc show ChIP-seq data as read coverage. We prefer showing read coverage of IP data because it directly shows the level of background fragments in the IP samples, hence quality of the IP experiments. In the case of showing ChIP-seq data as log2 fold enrichment, normalization by the input visually lowers the background levels, and quality of the IP experiments cannot be directly evaluated.

We thank you for the adding the Venn diagram which shows an overlap of peaks between the two ChIP-seq experiments. While we understand your justification for presentation of data, if your data analysis strictly follows ENCODE guidelines (for coverage, quality, and reproducibility), visual analysis of the quality of IP experiments should not be necessary. If you prefer to show read coverage of your IP samples, please include the read coverage from the corresponding input in each panel so that the quality of the IP experiments can be directly evaluated as you suggest.

22

***15. The way the authors showed the overlap between the increased and decreased transcripts between AP2-FG2 and AP2R-2 in Figure 6E with a correlation plot is not appropriate for the distribution of the data. For a linear correlation in the data to be drawn, the data needs to follow a linear distribution, which is not the case for their increased and decreased transcripts. The authors can simply show what proportion of transcripts are differentially abundant in each of the conditions.***

Thank you for your suggestion. Since we proved that *Pb*AP2-FG2 and *Pb*AP2R-2 form a complex by the RIME (thanks to all three reviewers’ suggestions), we think that the data showing linear correlation between log2(fold change) of *pbap2-fg2*(-) vs WT and *pbap2r- 2*(-) vs WT, which was only an indirect proof for association of *Pb*AP2-FG2 and *Pb*AP2R-2, can be omitted. However, we are not sure how the analysis was not appropriate, reading your statement that “For a linear correlation in the data to be drawn, the data needs to follow a linear distribution, which is not the case for their increased and decreased transcripts.” We performed linear approximation (actually linear regression) to show how much the data can fit to a linear equation with R-squared. If necessary, we could provide *t*-value and *p*-value for a slope of the regression equation, which were 30 and 1.8 × 10^-90^, respectively. We would really appreciate if you could teach us textbooks or studies that explain your statement in detail, and that will be considerably helpful for us to improve our future studies.

Thank you for attempting to address our initial comment regarding the correlation plot displayed as Figure 6H. We apologize for not being clearer in our original request. There are a couple of reasons that this analysis is statistically inappropriate. 1) The data points on the graph are not calculated entirely independent of each other. For the FC calculation, you do not have an independent WT measurement for each knockout sample. Each WT timepoint is used in the calculation of one of each knockout and, therefore, the Log2FC calculations are not completely independent of each other, and linear regression/approximation is not appropriate. (For example: What you are currently doing is A/B to C/B, where A and C are your knockout strains and B is the WT). The correct comparison would be A/B to C/D OR directly A to C. 2) The data are already filtered for statistical significance resulting in two “clusters” of data points and skewing the data toward a linear relationship based on the extreme fold changes.

There are a few things that can be done to make your analysis appropriate:

1) To draw a statistical conclusion from a correlation plot all of the data should be independent of each other. For instance, since your denominator for the Log2FC calculation per gene is the same across the knockout genotypes, you might consider just plotting the average expression value per gene with each knockout represented on a different axis, followed by a linear regression/approximation analysis. One can use a density correlation plot for large data sets.

2) Alternatively, the Log2 expression of significant genes in either knockout line can be represented on separate graphs as a box plot where the WT expression of select significant genes is separate from your knockout expression and the upward or downward trend will support/refute the hypothesis.

3) More simplistically, you could represent the significantly up or downward FC of sets of genes in both knockout strains as a Venn Diagram to demonstrate the overlap of regulated genes.

***16. Please scale ChIP-seq tracks between groups and include y- and x-axes on ChIP- seq tracks for figures 1,3,6 and 7. When showing ChIP-seq tracks also clarify whether what is being displayed is truly “coverage” or actually log2(ChIP/input).***

Sorry for the lack of information in the figures showing ChIP-seq tracks. We described y- and x-axis scales in the figures you mention. We also described that the read coverage for ChIP data was shown in these figures in the Figure captions.

We thank you for the adding the clarification to the figures and corresponding captions.

***17. In Supplementary Figure 1: the authors should state which primer sets illustrated in the pictograms produced the PCR products shown in the figure and the conditions of the PCR should be stated: nr of cycles, annealing temperature of primers should be included in supplementary table S8.***

Thank you for your suggestion. We modified S1 Fig (S2 Fig in the revised manuscript) accordingly and described conditions of the PCR in the Materials and Methods as follows; Line 705-707. “The PCRs were performed using PrimeSTAR GXL DNA Polymerase (Takara). PCR products were amplified for 30 cycles, and annealing temperature was set at 55 °C.”

Since we employed the same conditions for all PCR experiments, including those for donor construction and genotyping, we indicated this information on the top of S8 table instead of stating for each primer.

We thank you for the adding the experimental details to this supplemental figure.

***18. Line 455-457: The authors state they set their FPKM threshold at <10, but it seems they meant to include transcripts with FPKM >10.***

Thank you for your comment. As you suggest, we meant that we have included transcripts with FPKM > 10 in the analysis. We removed “>” to make the statement correct.

I think this error still exists in the manuscript. See line 547, “setting a minimum FPKM threshold of < 10” should read “threshold of FPKM ≥ 10”. Please ensure that you have accurately represented the thresholds you used throughout the study.

***19. Please revise or complete the following requests for the Materials and Methods section of the paper:***

***a. Please include a detailed method for the “fluorescence analysis” of the parasites- including what microscope was used, if this was an immunofluorescence assay or live fluorescence microscopy etc.***

We added the “fluorescence analysis” method in the Materials and Method section as follows;

Line 710-713. “Expression of GFP-fused *Pb*AP2-FG2 was analyzed by live fluorescence microscopy. Cells were stained with 1 ng/mL Hoechst 33342 for 10 min. Images were taken by Olympus BX51 microscope with Olympus DP74 camera.”

Thank you for the above revision.

***b. For the generation of plasmid for transgenic alteration of the parasite, include more details on the plasmid background, with appropriate references, should be stated clearly, with a higher level of detail than simply naming them: ie. “gfp-fusion vector” and “targeting construct”***

We are sorry that description of plasmid constructs was insufficient in the manuscript. We rewrote the methods as follows and added a schematic illustration of *gfp*-fusion vector in S2A Fig;

Line 680-691. “Briefly, for *gfp*-tagging, two homologous regions were cloned into the *gfp*-fusion vector to fuse *pbap2-fg2* in-frame with *gfp*. The *gfp*-fusion vector was previously generated on the backbone of pBluescript SK (+) (Stratagene, La Jolla, CA, USA). In this vector, 3’ untranslated region (UTR) of *hsp70* and a *hdhfr* expression cassette (under the control of *pbef-1α* promoter and 3’ UTR of *pbdhfr*) were located 3’- side of the *gfp* gene in this order. Of the two homologous regions, one was cloned to 5’- side of the *gfp* using *Xho*I/*Nhe*I site, and the other was cloned to 3’-side of the *hdhfr* cassette using *Bam*HI/*Not*I site (S2A Fig). The plasmid was linearized by *Xho*I and *Not*I digestion before use in transfection experiments. To knock out *pbap2-fg2*, the targeting construct was prepared using overlap PCR. The construct had two homologous regions around the *pbap2-fg2* locus flanking a *hdhfr* expression cassette, which was derived from the *gfp*-fusion vector described above.”

Thank you for the above revision.

***c. The authors stated the parasite lines were verified by PCR and/or Sanger sequencing. Please specifically state which methods were used in which cases, if Sanger sequencing was used to verify these lines please provide supplementary data to this effect.***

Sorry for our unclear statement. We only performed Sanger sequencing for the motif_mutated parasite, the result of which was provided in Fig 5A. To clarify, we made a change in the sentence as follows;

Line 704-705. “Recombination was confirmed by PCR and, for the motif_mutated parasite, Sanger sequencing.”

Thank you for the above revision.

***d. The authors should consider performing next-generation whole genome sequencing on the knockout parasite lines to verify that there are no other mutations that could contribute to the phenotype.***

Thank you for your comment. We think that performing NGS of the whole genome every time someone develops a knockout line seems too much work for a phenotype analysis. To exclude possibility of existence of other mutations that contribute to a certain phenotype, we assess two independent clones for each knockout experiment.

Thank you for the above revision. However, with the cost of Next generation sequencing becoming much more affordable, whole-genome sequencing of each strain created should be carried out to demonstrate that there are no additional mutations that exist within the parental or newly genetically modified lines. Assessing two clones does not necessarily preclude the existence of additional mutations that may arise in combination with the genetic disruption. I would think it would be advantageous for you to investigate the genome sequence thoroughly.

***e. For the ChIP-seq experiments- there seems to be substantial information about the experimental protocol missing without any supplementary method being cited. Please provide a more detailed protocol and ensure to include the following detail OR cite the source of the protocol that includes these details: Was a nuclear extraction performed prior to sonication? What is the composition of the SDS lysis buffer? At which temperature were the samples sonicated? What was the concentration of anti-GFP antibodies used in the ChIP-seq experiment and how long was the antibody incubation performed? Were the lysates precleared before the immunoprecipitation step? When were the input control samples taken? The authors also don’t mention a proteinase digestion step, was this omitted? How was the immunoprecipitated DNA purified?***

We are sorry for the lack of information in our ChIP-seq method. We rewrote the method as follows;

Line 735-753. “Whole blood was withdrawn from the infected mice treated with sulfadiazine and passed through the Plasmodipur filter to remove white blood cells. The blood was diluted in a complete medium (RPMI1640 supplemented with 20% fetal calf serum) and immediately fixed with 1% formalin at 30 °C. After fixing, RBCs were lysed in ice-cold 1.5 M NH4Cl solution. This step was performed several times until the supernatant became clear, and the cells were lysed in SDS lysis buffer (50 mM Tris-HCl, 1% SDS, 10 mM EDTA). The samples were sonicated at 4 °C using Bioruptor (Cosmo Bio) for 20 cycles of 30 sec on/30 sec off to shear the chromatin. Input samples were collected at this point from the sonicated cell lysate. For IP samples, chromatins were immunoprecipitated with anti-GFP polyclonal antibodies (5 mg/mL; Abcam, ab290), which were bound to Protein A Magnetic Beads (Invitrogen) before the ChIP step (2 μL of antibody was mixed with 20 μL of beads and incubated for > 1 h). After 12 h of incubation at 4 °C, the beads were washed with low-salt buffer (20mM Tris-HCl, 0.1% SDS, 1% Triton X-100, 2 mM EDTA, 150 mM NaCl) for five times and high-salt buffer (20mM Tris-HCl, 0.1% SDS, 1% Triton X-100, 2 mM EDTA, 500 mM NaCl) for three times. Immunoprecipitated chromatin was then eluted in elution buffer (10mM Tris-HCl, 1% SDS, 5 mM EDTA, 300 mM NaCl), heated at 65 °C for 8 h, and processed with RNase H for 1 h and proteinase K for 2 h. DNA fragments were purified from the immunoprecipitated chromatin by phenol/chloroform extraction and ethanol precipitation and used for library construction.”

To clarify, we did not perform nuclear extraction or preclearance of cell lysate with antibody-free protein A beads.

Thank you for the above revision.

***f. There is also information missing regarding the data analysis of the ChIP-seq experiments: What version of the Plasmodium berghei genome was used to map the sequencing reads? How/why did the authors decide on a fold change enrichment threshold of >3?***

The genome sequence data was version 3 obtained from PlasmoDB 46. We added this information in the Materials and Methods. Thank you for the notice.

About decision of a fold enrichment threshold for ChIP-seq analyses, most studies set the threshold between 2 and 4 without providing any particular reason why the value was selected, to the best of our knowledge. Thus, we think setting the fold enrichment threshold as 3 is fairly standard. In the present manuscript, we preferred the value for several reasons, including overlap of peaks in duplicates and ratio of peaks with the enriched motifs. Just to be clear, we used the criteria provided in the *P. yoelii* study, in which a fold enrichment threshold was 2, to reanalyze the ChIP-seq data of *Py*AP2-O3 because we thought that would be fair to the previous study.

Thank you for the above revision and clarification as to your choices regarding fold change enrichment cutoffs.

***g. For the ChIP-qPCR and RT-qPCR experiments, what were the starting concentrations of DNA or RNA included in the experiments respectively and how many cycles were these experiments conducted for? Did the authors include reference genes for either study for normalization? No account is given of how these data were analyzed, was the 2-delta Ct method used for quantification in the qPCR?***

For the ChIP-qPCR analysis, we did not quantify the concentration of DNA samples. Since IP and input samples were harvested from 200 μL and 10 μL of the cell lysate, respectively, the % input values were simply calculated as 2^(Ct^IP^-Ct^input^) × (1/20) × 100 without normalization by reference genes. For the RT-qPCR, cDNA was synthesized from 1 μg of total RNA, and 1/200 of the synthesized cDNA was used for qPCR. Amplification step of qPCR was conducted for 40 times, and Ct values ranged from 20 to 35. We described these details as follows;

Line 774-785. “IP and input samples were each prepared from 200 μL and 10 μL of sonicated cell lysate, respectively. Quantification of DNA fragments of interest was performed by real-time qPCR using TB Green Fast qPCR Mix (Takara) and Thermal Cycler Dice Real Time System II (Takara). Amplification cycles were performed for 40 cycles, and cycle threshold (Ct) was detected between 20 to 35 cycles. From the Ct values, %input values were calculated as 2^(Ct^IP^-Ct^input^) × (1/20) × 100. Three biologically independent experiments were performed and used for the analysis.

For RT-qPCR analyses, cDNA was synthesized from 1 μg of total RNA, extracted as described for RNA-seq analysis, using the PrimeScript RT reagent Kit with gDNA Eraser (Takara), and 1/200 of the synthesized cDNA was used for qPCR. Real- time qPCR experiments were performed as described above.”

Thank you for the above revision and clarification.

***20. Why did the authors use the Fisher’s exact test to analyze enriched motifs in 6 bp bins instead of using available softwares (ie STREME) that scan over a wider range? Could you please justify your use of the Fisher’s exact test in this context?***

We perform enrichment analysis using the Fisher’s exact test on all motifs, so that all candidates of possible binding motifs can be detected. In addition, because enrichment of a motif in peak regions is analyzed against the other genomic regions, sequence bias in the genome can be considered in this analysis. In the present study, we only showed enrichment analysis of 6-bp motifs, but usually, we perform the analysis on motifs with various length, mostly from 5-bp to 10-bp. We chose the length considering their *p*-values and how many peaks contain the motif within the peak region.

We agree that currently available software, such as MEME and STREME, is also useful. However, sometimes these methods produce incorrect binding motifs, which has been seen in the Py study, for some reasons. We suppose that how you prepare control sequences somehow affects the result. We think that our analysis is more thorough and simpler and hence able to discover a true binding motif although the unification of enriched motifs needs to be done manually.

Thank you for the above clarification.

***21. How did the authors associate genes with Chip-seq peaks if the binding site overlaps within 1200 bp of the start codon? This method does not take into account which genes are closest to the binding site or whether these bound regions are intra- or intergenic.***

In our analysis, we actually measured distance between peak summit and a start codon for each gene. However, we decided to include both genes in the targets if a peak located within 1200 bp of two genes because bidirectional promoters are often observed in the

*Plasmodium* genome, especially for small intergenic regions. In addition, because the

distance between TSS and start codon varies depending on genes, not always the closer gene is the true target for a binding site. To include as many true targets as possible, we do not consider the distance a peak summit and a start codon for target analysis.

Targets were predicted from peaks on intergenic regions, and any peak on intragenic regions was not used for target prediction. The “within 1200 bp” threshold is only set for genes whose upstream intergenic region was larger than 1200 bp. To clarify this method in the manuscript, we made a change in the Results section as follows;

Line 229-232. “The analysis revealed that of the 638 highly reproducible peaks, 558 peaks located in intergenic region. These intergenic peaks located in the upstream regions (within 1200 bp from ATG) of 517 genes, suggesting that these genes are a target gene of

*Pb*AP2-FG2 (S3C Table).”

Thank you for the above clarification and revision to the text.
