# Supplementary material for: PbAP2-FG2 and PbAP2R-2 function together as a transcriptional repressor complex essential for Plasmodium female development
Source: PLoS Pathog. 2023 Feb 13;19(2):e1010890. doi: 10.1371/journal.ppat.1010890 (PMC9956629; doi:10.1371/journal.ppat.1010890)
Supplement: Attachment — Submitted filename: Responce_Letter2.docx [file ppat.1010890.s016.docx]

2/1/2023

Responses to Reviewers:

**Part I – Summary**

***Reviewer #1: This is a timely and well-done study. The authors responded to all the points this reviewer raised. The paper was significantly improved with the addition of new controls and complementary experiences.***

Thank you so much for your time and effort on reviewing our manuscript. Thanks to your previous review and comments, our manuscript has been significantly improved.

***Reviewer #2: In their manuscript: “PbAP2-FG2 and AP2-R2 function together as a transcriptional repressor complex essential for Plasmodium female development”, Nishi et al. characterize the potential transcriptional regulatory role of two ApiAP2,***

***DNA-binidngproteins, AP2R-2 and AP2-FG. Through phenotypic analysis it would seem the AP2-FG2 protein peaks in expression in female gametocytes and the genetic perturbation of this protein results in a decrease in female gametocyte-related genes. The protein seems to exert its function through binding upstream regions of genes,***

***repressing male and female-related gametocyte genes alike.***

***The authors were given review comments and I appreciate the authors efforts to address the concerns I had with their study following the initial submission. Most comments/concerns were adequately addressed and the addition of RIME to determine the interacting partners of AP2-FG2 strengthens this study. I am, however, still curious about the underlying AP2-FG2 GFP-tagged line. I have commented on the authors responses in line in the attached document and summarized below in the "Minor Issues" section***

We would like to thank you for your time and effort. Thanks to your review and comments, our manuscript has been significantly improved. We sincerely revised our manuscript and provided responses to your comments below.

***Reviewer #3: Overall the authors have addressed all of my major concerns. I have some additional suggestions and minor points raised below.***

We would like to thank you for taking your time and effort. Your review and comments considerably helped us to improve our manuscript. We sincerely revised our manuscript and provided responses to your comments below.

**Part III – Minor Issues: Editorial and Data Presentation Modifications**

***Reviewer #2: In response to Reviewer #2 Comment 2, the authors suggest that the GFP-signal does not need to be quantified or more clearly demonstrated to be exclusive to female gametocytes. I think it is good scientific practice to confirm any imaging with Western Blot analysis. Part of establishing confidence in the genetic line that has been created and reagents used in the ChIP-seq is first proving that the full-length GFP-tagged PbAP2-FG2 protein is exclusively expressed in the female gametocyte and that it is cleanly identified by the antibody used for the ChIP-seq by showing a Western Blot.***

Thank you for your comment. While we understand that Western Blot analysis could confirm protein expression observed in fluorescent analysis and also expression of full-length protein, we still think that it is not absolutely necessary. In addition, it is technically very difficult to detect a transcription factor by Western Blot using male and female gametocytes separated by a cell sorter because number of cells that can be harvested by cell sorting is limited, and transcription factors are generally not expressed at a high level. For ChIP-seq reagents, we have confirmed that they are well suited through several other ChIP-seq experiments. Meanwhile, we would like to consider your suggestion about Western Blot analysis for our future studies.

***In response to Comment 13, I thank the authors for performing the additional RIME experiment that empirically establishes the interaction of PbAP2-FG2 and PbAP2R-2. Could the authors speculate as to why AP2-I, AP2-G2 and AP2-O2 are also found to complex with PbAP2-FG2? This should be added to the discussion of the results of RIME. Additionally, please discuss your results with respect to the recently published P. berghei male/female gametoycte differentiation and maturation ssRNA-seq study https://doi.org/10.1016/j.chom.2022.12.011.***

Thank you for your comment. For *Pb*AP2-O2, we have already discussed its possible role as a transcriptional repressor in the Results section. For *Pb*AP2-I and *Pb*AP2-G2, we did not provide any discussion, because their quantitative values were not high in the *Pb*AP2-FG2::GFP samples, and hence we were not confident that they were an interaction partner of *Pb*AP2-FG2.

Thank you for your suggestion about making discussion regarding on the recent study by Russel et al. We added the following sentence in the Discussion section;

Line. “Very recently, Russel et al. identified five genes that are essential for male differentiation [45], but these genes were not included in the target gene list of *Pb*AP2-FG2 and *Pb*AP2R-2.”

***In response to Comment 14, I thank you for the adding the Venn diagram which shows an overlap of peaks between the two ChIP-seq experiments. While we understand your justification for presentation of data, if your data analysis strictly follows ENCODE guidelines (for coverage, quality, and reproducibility), visual analysis of the quality of IP experiments should not be necessary. If you prefer to show read coverage of your IP samples, please include the read coverage from the corresponding input in each panel so that the quality of the IP experiments can be directly evaluated as you suggest.***

Thank you for your comment. We added heatmaps for input samples as supplementary figures.

***In response to Comment 15, thank you for attempting to address our initial comment regarding the correlation plot displayed as Figure 6H. WI apologize for not being clearer in the original request. There are a couple of reasons that this analysis is statistically inappropriate. 1) The data points on the graph are not calculated entirely independent of each other. For the FC calculation, you do not have an independent WT measurement for each knockout sample. Each WT timepoint is used in the calculation of one of each knockout and, therefore, the Log2FC calculations are not completely independent of each other, and linear regression/approximation is not appropriate. (For example: What you are currently doing is A/B to C/B, where A and C are your knockout strains and B is the WT). The correct comparison would be A/B to C/D OR directly A to C. 2) The data are already filtered for statistical significance resulting in two “clusters” of data points and skewing the data toward a linear relationship based on the extreme fold changes.***

***There are a few things that can be done to make your analysis appropriate:***

***1) To draw a statistical conclusion from a correlation plot all of the data should be independent of each other. For instance, since your denominator for the Log2FC calculation per gene is the same across the knockout genotypes, you might consider just plotting the average expression value per gene with each knockout represented on a different axis, followed by a linear regression/approximation analysis. One can use a density correlation plot for large data sets.***

***2) Alternatively, the Log2 expression of significant genes in either knockout line can be represented on separate graphs as a box plot where the WT expression of select significant genes is separate from your knockout expression and the upward or downward trend will support/refute the hypothesis.***

***3) More simplistically, you could represent the significantly up or downward FC of sets of genes in both knockout strains as a Venn Diagram to demonstrate the overlap of regulated genes.***

Thank you so much for thoroughly explaining the issue in our statistical analysis for Fig 6H. We now understand why our analysis for comparing the fold change values between *pbap2-fg2*(-) vs WT and *pbap2r-2*(-) vs WT was inappropriate. Following your advice (1), we constructed a new plot which shows log_10_(RPKM) value in *pbap2-fg2*(-) against that in *pbap2r-2*(-) for all genes. A linear approximation based on this plot depicted a line with a slope of 1.07 and a mean square correlation coefficient of 0.92. Accordingly, we modified the Results section as follows;

Line . “To further evaluate the relationship between *pbap2-fg2*(-) and *pbap2r-2*(-) in detail, we plotted RPKM value in *pbap2-fg2*(-) against that in *pbap2r-2*(-) for each gene. We then constructed a linear approximation of the plots, which depicted a line with a slope of 1.07 and a mean square correlation coefficient of 0.92 (Fig 6H).”

We sincerely appreciate your teaching and will make use of it for our future studies as well.

***Reviewer #3: In the revised manuscript the authors now provide evidence for a direct interaction between PbAP2-FG2 and PbAP2-R2 using Rapid Immunoprecipitation Mass Spectrometry of Endogenous Proteins (RIME), which combined ChIP and MS to identify associated proteins. Although using an unrelated GFP-tagged line to monitor unspecific interactions would have been a more appropriate negative control for this experiment, the pull-down of PbAP2-FG2 detected PbAP2-R2 demonstrating that PbAP2-FG2 and PbAP2-R2 likely interact. Other proteins shown in Figure 7 appear to be more hand selected from their IP data (based on examination of Table S6), and I am less convinced by the results based solely on enrichment (over the WT control), such as PbMORC and other chromatin remodeling complexes, although these are interesting observations.***

Thank you for your comment. We think an unrelated GFP-tagged line would not be an appropriate negative control because proteins IPed in such a condition cannot be distinguished whether they derived from unspecific interaction or are specifically IPed for the unrelated GFP-tagged protein. In contrast, using the WT control would identify proteins unspecifically interacted with anti-GFP antibody or protein A beads. Just for your information, we have already performed RIME for other factors at the gametocyte stage and did not detect *Pb*AP2-FG2, *Pb*AP2R-2, or *Pb*MORC etc.

Enrichment over a control sample is a criterion that is used in normal IP-MS analysis as well. Therefore, we think that the proteins listed in Fig 7 should be considered as a possible interaction partner of *Pb*AP2-FG2.

***A few additional minor points:***

***Line 21 of the abstract should read: “Despite the number of studies on gametocyte development that have been conducted, the molecular mechanisms regulating this process remain to be fully understood.”***

Thank you for your comment. We modified the sentence accordingly.

***If PbAP2-FG2 and PbAP2R-2 function together, why does the abstract only mention “a significant overlap between the target genes of PbAP2-FG2 and AP2-G.” Why not also add PbAP2R-2 since these two proteins overlap in target gene specificity?***

Thank you for your comment. As you mention, *Pb*AP2R-2 targets also overlap with *Pb*AP2-G targets. However, technically, what we have performed in the Results was comparison between the target genes of *Pb*AP2-FG2 and *Pb*AP2-G. Therefore, we mentioned “Comparative target analysis between *Pb*AP2-FG2 and *Pb*AP2-G demonstrated a significant overlap between their target genes, suggesting that repression of early gametocyte genes activated by *Pb*AP2-G is one of the key roles for this female transcriptional repressor complex.”

***Should PbAP2R-2 be renamed PbAP2-FG3? Why or why not?***

Thank you for your suggestion. Although we have stated that *Pb*AP2R-2 have an AP2-like domain, we are not sure if it is actually an AP2 domain. We thought that we should not name it as an AP2-family protein unless its direct interaction with DNA was proven perhaps by EMSA or DIP. Therefore, we kept the name *Pb*AP2R-2, which derives from AP2 TF-related gene 2.

***Line 111, please change “AP2-family transcription factor” to “ApiAP2-family transcription factor”***

Thank you for your comment. However, we would like to keep the phrase unchanged unless it causes a huge problem to readers. We think that Apicomplexan AP2 proteins are not very distinguished from the other AP2 proteins as no protein domain database distinguish them.

***The authors should modify their citation of Russell et al. since it has now been published in Cell Host & Microbe and is no longer solely a bioRxiv preprint.***

Thank you for your kind notice. We modified the citation accordingly and removed the phrase “(still in a preprint)”.

***The authors have added additional support for the presence of an AP2 domain (at least one) in PbAP2-R2 (lines 340-346). Please include alignments of the regions that they find to not be well-conserved between Pb and Pf as supplemental data. Also, I would again encourage the authors to use AlphaFold2, which clearly demonstrates at least one well-folded AP2 domain. Given that this protein is likely to contain at least one AP2 domain, it would be good to speculate what the role of this AP2 domain might be given that the binding matches that of PbAP2-FG2?***

Thank you for your comment. We provided alignments of the two regions of AP2R-2 mentioned in the manuscript according to your suggestion. About AlphaFold2, we performed the prediction by the program for the previous response (stated as follows: “With the N-terminal adjacent region, we predicted 3D structure of this conserved region (position 1202-1257), which resulted in a structure similar to AP2 with the first beta-sheet not formed. However, the 3D structure was not found similar to any AP2 by VAST search, and this region did not fully contain consensus amino acids for AP2.”). We are sorry that we did not clearly state that we used AlphaFold2. For the VAST search, we used the PDB file produced by the AlphaFold2. Colocalization of *Pb*AP2-FG2 and *Pb*AP2R-2 was due to the formation of a complex between the two factors as proven by the RIME analysis. Thus, we do not think that these factors independently interacted with DNA using their own DNA binding domain.

***Finally, I have an alternative title suggestion: “PbAP2-FG2 and AP2R-2 function together as an essential transcriptional repressor complex during female gametocyte development in Plasmodium”***

Thank you for your suggestion. However, we would like to keep the title unchanged because the other two reviewers accepted the current title.
